# Pharmacometabolomics Enables Real-World Drug Metabolism Sciences

**DOI:** 10.3390/metabo15010039

**Published:** 2025-01-10

**Authors:** Fleur B. Nijdam, Marieke A. J. Hof, Hans Blokzijl, Stephan J. L. Bakker, Eelko Hak, Gérard Hopfgartner, Frank Klont, on behalf of the TransplantLines Investigators

**Affiliations:** 1 Unit of PharmacoTherapy, Epidemiology and Economics, Groningen Research Institute of Pharmacy, University of Groningen, Antonius Deusinglaan 1, 9713 AV Groningen, The Netherlands; f.b.nijdam@rug.nl (F.B.N.); e.hak@rug.nl (E.H.); 2 Department of Analytical Biochemistry, Groningen Research Institute of Pharmacy, University of Groningen, Antonius Deusinglaan 1, 9713 AV Groningen, The Netherlands; m.a.j.hof@rug.nl; 3 Department of Gastroenterology, University Medical Center Groningen, University of Groningen, Hanzeplein 1, 9700 RB Groningen, The Netherlands; h.blokzijl@umcg.nl; 4 Division of Nephrology, Department of Internal Medicine, University Medical Center Groningen, University of Groningen, Hanzeplein 1, 9700 RB Groningen, The Netherlands; s.j.l.bakker@umcg.nl; 5 Life Sciences Mass Spectrometry, Department of Inorganic and Analytical Chemistry, University of Geneva, Quai Ernest Ansermet 24, 1211 Geneva, Switzerland; gerard.hopfgartner@unige.ch; 6 Department of Clinical Pharmacy and Pharmacology, University Medical Center Groningen, University of Groningen, Hanzeplein 1, 9700 RB Groningen, The Netherlands; 7 Group of Authors on Behalf of the Transplant Lines Biobank and Cohort Study, University Medical Center Groningen, University of Groningen, Hanzeplein 1, 9700 RB Groningen, The Netherlands; transplantlines@umcg.nl

**Keywords:** personalized medicine, pharmacogenomics, pharmacometabolomics, real-world, drug metabolism, human

## Abstract

**Background/Objectives**: Pharmacogenomics (PGx) has revolutionized personalized medicine, notably by predicting drug responses through the study of the metabolic genotype of drug-metabolizing enzymes. However, these genotypes rely heavily on the availability and completeness of drug metabolism information and do not account for (all) “phenoconversion” factors, like drug–drug interactions and comorbidities. To address these limitations, a more phenotypic approach would be desirable, for which pharmacometabolomics (PMx) could be useful by studying and elucidating drug metabolism in patient samples, such as blood and urine. **Methods**: This study explored the potential of PMx to analyze real-world drug metabolite profiles of the extensively studied drug cyclosporine (CsA) using 24-h urine samples from 732 kidney and 350 liver transplant recipients included in the TransplantLines Biobank and Cohort Study (NCT identifier NCT03272841). Detected metabolites were matched with existing information on CsA metabolism gathered through a comprehensive literature review, aiming to confirm previously reported metabolites and identify potentially unreported ones. **Results**: Our analyses confirmed the urinary presence of CsA and six known metabolites. Additionally, we detected three known metabolites not previously reported in urine and identified one unreported metabolite, potentially suggesting the involvement of glutathione conjugation. Lastly, the observed metabolic patterns showed no notable differences between kidney and liver transplant recipients. **Conclusions**: Our findings demonstrate the potential of PMx to enhance the understanding of drug metabolism, even for well-studied compounds such as CsA. Moreover, this study highlights the value of PMx in real-world drug metabolism research and its potential to complement PGx in advancing personalized medicine.

## 1. Introduction

Personalized medicine has revolutionized the field of healthcare, enabling treatment approaches tailored to individual patient characteristics [1]. This field is largely driven by technological advancements, for example, leading to the clinical implementation of genetic screening methods to study variations in the genes encoding drug-metabolizing enzymes, drug transporters, and drug targets, as they may influence an individual’s drug response [2]. Pharmacogenomics (PGx) has emerged as a powerful tool in this regard, notably studying the metabolic genotype of drug-metabolizing enzymes in an individual to predict whether someone has a poor, intermediate, normal, rapid, or ultrarapid metabolizer phenotype [3,4]. This genotyping helps predict therapeutic response, allowing for more precise dosing and more effective drug treatments, as well as a reduction in adverse drug reactions [5].

A key feature of PGx is its reliance on information about how and by which enzymes a drug is metabolized. It is, however, often overlooked that this information is generally derived exclusively from so-called mass balance studies, which are small-scale drug metabolism studies conducted in a few healthy male volunteers receiving a single dose of the target drug [6]. Therefore, it may not be surprising that recent “real-world” drug metabolism studies have described various previously unreported metabolites for drugs that have been on the market for decades [7,8].

These real-world drug metabolism studies utilized the pharmacometabolomics (PMx) technique to investigate drug metabolism in large patient populations. More specifically, the PMx technique employed in these studies is an extension of the pioneering work of Kaddurah-Daouk [9] and Everett and Nicholson [10] within the PMx field, which represents a subdomain of metabolomics that studies endogenous metabolite profiles in relation to drug therapies. Recent advances in PMx have highlighted its ability to characterize baseline metabolic profiles, identify metabolite biomarkers for drug efficacy, and uncover metabolic pathways associated with individual drug responses. By leveraging endogenous metabolite data, PMx has also facilitated the prediction of adverse drug reactions and the stratification of patients based on their metabolic phenotypes, providing actionable insights in the context of precision medicine [11,12,13,14,15]. The recent studies of Klont et al. [7,8] furthermore extended the focus to exogenous metabolites, as can often be studied using the same metabolomics datasets, though typically necessitating different data-processing strategies [16]. This PMx workflow, therefore, provides a more comprehensive understanding of how drugs are metabolized on an individual level, yielding a more phenotypic view of drug metabolism and potentially bringing interindividual differences in drug metabolism to light.

The studies by Klont et al. [7,8] admittedly did not involve an exhaustive search for all possible metabolites (above a pre-set intensity threshold). Hence, the discovery of previously unreported metabolites was arguably coincidental rather than a primary objective. Assessing the potential clinical usefulness of PMx and its complementarity to PGx, thus, requires a more systematic approach. In this regard, an ideal model compound for such a study is the therapeutic drug cyclosporine (CsA). This immunosuppressant has been studied extensively in the past decades, providing a comprehensive overview of CsA metabolites in bile, blood, and urine [17,18,19]. In addition, recent findings suggest the presence of various CsA-related signals of very high abundance in urine samples from CsA users, which seems counterintuitive given that this drug is primarily excreted in feces [20]. A PMx study targeting this drug could, thus, demonstrate its discovery potential by confirming previously identified metabolites and potentially uncovering novel insights into CsA metabolism.

In this work, we investigate the potential of PMx (in this project using a “SWATH” mass spectrometry-based workflow) to analyze real-world drug metabolite profiles of the well-studied immunosuppressant CsA in 24-h urine from a large group of human liver and kidney transplant recipients (historically being one of the largest populations using this drug) participating in the TransplantLines Biobank and Cohort Study [21]. Firstly, we aim to explore existing knowledge of CsA metabolism and its metabolites through a comprehensive literature review. Secondly, we aim to confirm the presence of the known CsA metabolites and potentially unveil previously unreported CsA metabolites in urine samples (as level 3 “putatively characterized compound classes”, according to the Metabolomics Standards Initiative, MSI [22]).

## 2. Materials and Methods

### 2.1. A Literature Study of CsA Metabolites

A comprehensive literature review was conducted on PubMed to find relevant articles that reported CsA drug metabolism studies. An overview of the search strategy can be found in Method S1. Original research articles written in English were included in this review. Other article types, such as review articles, meta-analyses, case reports, book sections, protocols, commentaries, editorials, and letters to the editor, were excluded. Exclusion criteria for original research articles consisted of articles that did not address CsA metabolism, described in vitro CsA metabolism, performed CsA metabolism studies in animals, and articles that were not related to CsA in any form. Titles, abstracts, and, if needed, full texts were retrieved and screened. Screening and study selection were carried out using EndNote 21 [23]. A PRISMA flow diagram shows the inclusion criteria of this literature study (see Figure S1).

### 2.2. Clinical Samples

Twenty-four-hour urine samples were available for kidney (KTR) and (potential) liver transplant recipients (LTR) included in the TransplantLines Biobank and Cohort Study (NCT identifier NCT03272841). This study was approved by the Institutional Review Board of the University Medical Center Groningen (UMCG; decision METc 2014/077) and adheres to the Declaration of Helsinki, the Declaration of Istanbul, and the UMCG Biobank Regulation [21]. The urine samples were collected per strict protocol, which was designed internally for generic biobanking purposes and lacked the addition of preservative agents commonly used in metabolomics research. For sample collection, BD Vacutainer 24-h urine collection containers were used, and the time between sample collection and handing it in was consistently below 48 h. Samples were subsequently stored at −20 °C for up to four days after manual aliquoting and at −80 °C and atmospheric pressure for up to five years until shipment (<72 h on dry ice in a security-sealed, insulated box compliant with IATA, ADR, and 49 CFR (DOT) transport regulations). Finally, samples were stored at −80 °C and atmospheric pressure for up to six months after shipment prior to analysis. For this pharmacometabolomics study, we analyzed samples from 570 KTR who were ≥1 year post-transplantation and had already been transplanted prior to the start of the TransplantLines study (study A), 163 KTR who were followed prospectively and for whom samples were available at 3, 12, and 24 months post-transplantation (study B), 316 LTR who were ≥1 year post-transplantation and had already been transplanted prior to the start of the TransplantLines study (study C), and 176 (potential) LTR who were followed prospectively and for whom samples were available before transplantation and/or at 3, 6, 12, or 24 months post-transplantation (study D).

### 2.3. LC-SWATH/MS-Based Pharmacometabolomics Analyses

Urine samples were thawed (overnight at −25 °C, <4 h at 2–6 °C), vortex-mixed (30 s), and centrifuged (4 °C, 10 min, 14,000× *g*), after which 50 microliters of supernatant were transferred to glass inserts (BGB; Cat. No. 110501) placed in glass autosampler vials (BGB; Cat. No. SF2). Next, 10 microliters of a 5 pmol/µL internal standard solution in 10% methanol (see Table S1) were added to the samples; the vials were sealed with plastic caps (BGB; Cat. No. 070301), and the samples were vortex-mixed (30 s). From the resulting mixture, 24 microliters (≡20 microliters of urine, 20 pmol per internal standard) were analyzed by reversed-phase liquid chromatography coupled to high-resolution quadrupole-time-of-flight mass spectrometry operated in positive electrospray ionization and SWATH data-independent acquisition (DIA) modes. A detailed overview of LC and MS parameters is provided in Table S2, and more information on batch design and quality assurance is presented in Method S2, Figures S2–S13, and Table S3.

### 2.4. Data Processing

CsA-positive samples were identified by spectral library matching (SLM) [24] using SCIEX PeakView software (version 2.2.0.11391; 71 Four Valley Drive, Concord, ON, Canada, L4K 4V8) and an in-house generated reference spectrum for CsA (obtained with SCIEX TripleTOF instruments at a collision energy of 40 eV and a collision energy spread of 30 eV) (see Table S4 and Figure S14). Subsequently, an MS1-level feature-based evaluation of SLM results, as presented in [7], was employed to improve the reliability of the SLM results, for which the corresponding features were extracted using SCIEX MarkerView software (version 1.3.1; 71 Four Valley Drive, Concord, ON, Canada, L4K 4V8) (see Table S3). These same feature data were filtered based on *m*/*z* value (≥550) and retention time (≥14.0 and ≤15.5 min), thus zooming in on the regions where CsA and its metabolites were expected in our analytical study based on prior research findings [20]. Regarding the latter, filtering particularly simplified data analysis given the numerous high abundance signals present at lower retention times and originating from the polyethoxylated castor bean oil used in CsA capsules. Subsequently, the feature data were used to identify CsA-related signals by Mann–Whitney U test using CsA exposure status (exposed versus nonexposed, based on SLM findings) as a grouping variable and a *p*-value of <0.05 (which was Bonferroni-corrected) for assessing statistical significance. Next, significant hits were evaluated manually to exclude isotope peaks, adducts (e.g., sodium, ammonium), and low abundance features. Regarding the latter, a median abundance in CsA users of at least 1.0% relative to the highest observed median was used as a cut-off for inclusion. In addition, features with lower medians but showing a value of at least 5.0% (relative to the highest observed median) in at least one of the study samples were evaluated as well. Subsequently, representative samples were reanalyzed to yield “cleaner” fragment spectra by employing the product ion scan acquisition mode with narrower precursor isolation windows of 1 *m*/*z* unit (compared to window widths of 15 *m*/*z* units in the lower mass range and the two larger (>250 *m*/*z* units) windows in the higher mass range), while furthermore utilizing the same collision energy ranging from 10 to 70 V. In addition, more reliable signal intensities were extracted manually following an “SRM-like” targeted signal extraction approach [7] utilizing the SCIEX MultiQuant software (version 2.1) with a ±2.5 mDa mass extraction window and a 2.0-point Gaussian smoothing width. Finally, data analyses and computations were performed using R version 4.3.2 (R Foundation for Statistical Computing, Vienna, Austria). Subject characteristics are presented as median (interquartile range [IQR]) for continuous data and number with percentage (%) for categorical data. These characteristics were stratified by PMx-confirmed CsA use in both KTR and LTR separately and were investigated using the Mann–Whitney U test for non-parametric continuous variables, and the Chi-square test was applied to categorical variables. A *p*-value of <0.05 was considered statistically significant.

## 3. Results and Discussion

### 3.1. CsA Metabolites in Literature

**Figure 1:**
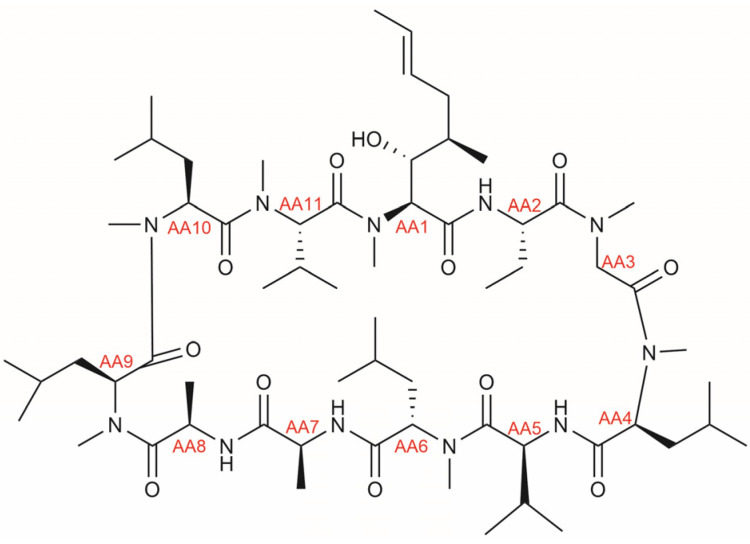
Chemical structure of cyclosporine A, with indication of amino acid (AA) positions shown in red.

The target drug CsA (see Figure 1) is a cyclic undecapeptide that is primarily cleared hepatically, although its metabolites have been found both in feces and urine (see Table 1). An early report detecting the presence of CsA in human samples (i.e., serum) was drafted by Yee et al. in 1982 using high-performance liquid chromatography (HPLC) [25], a technique that became critical for subsequent analyses of CsA and its metabolites in biological samples. Building on this, Maurer et al. [26] described nine metabolites of CsA in human urine, all representing CsA oxidation products having differentially hydroxylated (position 1, 6, 9), demethylated (position 4), and cyclized (position 1) amino acids. Also in urine, Meier et al. [27] detected a variant that was hydroxylated and featured a saturated double bond at the amino acid in position 1, and this variant was found in blood as well. Also in blood, Rosano et al. [28] confirmed the presence of CsA together with three metabolites previously found in urine [20], whereas Lensmeyer et al. [29] found six known and three additional oxidation products with differently oxidized amino acids (position 1, 4, 9) in blood, notably including a carboxylated variant.

The carboxylated metabolite had already been detected earlier in bile by Hartman et al. [30], together with unmetabolized CsA. In this same matrix, most of the metabolites previously found in urine were detected by several other studies conducted in the late 1980s and early 1990s [31,32,33], which additionally detected various unreported variants. Specifically, Wang et al. [31] detected a doubly hydroxylated (position 1, 9) variant featuring a saturated double bond at position 1, whereas Henricsson [32] and Christians et al. [33] detected phase II metabolites, respectively featuring a sulfate and glucuronide moiety at position 1. The latter group of authors also reported several previously unreported (phase I) metabolites but were generally unable to provide the exact positions of the added oxygens. Lastly, it should be acknowledged that some of the biliary metabolites have not (yet) been found in our target matrix (urine), but knowing about their existence will inevitably be helpful when detecting previously unreported metabolites.

**Table 1:** Overview of previously detected cyclosporine A metabolites sorted based on their molecular weight.

Substance Code	Molecular Formula	Monoisotopic Mass	Modification Reported on Position					Detected in		
			1	4	6	9	Unknown	Bile	Blood	Urine
AM4N	C_61_H_109_N_11_O_12_	1187.83		-CH_3_				[31,33]	[28,29]	[26]
CsA	C_62_H_111_N_11_O_12_	1201.84						[30,31]	[25,28,29]	[26]
AM4N9	C_61_H_109_N_11_O_13_	1203.82		-CH_3_		+OH		[33]	[29]	[26]
AM14N	C_61_H_109_N_11_O_13_	1203.82	+OH	-CH_3_				[33]	[29]	
*UM32.8min*	C_61_H_109_N_11_O_13_	1203.82	+OH	-CH_3_				[33]		
AM1AL	C_62_H_109_N_11_O_13_	1215.82	ald.					[33]		
AM1	C_62_H_111_N_11_O_13_	1217.84	+OH					[31,33]	[28,29]	[26]
AM1c	C_62_H_111_N_11_O_13_	1217.84	cycl.					[31,33]	[29]	[26]
AM9	C_62_H_111_N_11_O_13_	1217.84				+OH		[31,33]	[28,29]	[26]
AM4N69	C_61_H_109_N_11_O_14_	1219.82		-CH_3_	+OH	+OH		[33]		[26]
*UM21.2min*	C_61_H_109_N_11_O_14_	1219.82	+OH	-CH_3_			+OH	[33]		
AM1DI	C_62_H_113_N_11_O_13_	1219.85	+OH +sat.					[31]	[27]	[27]
AM1A	C_62_H_109_N_11_O_14_	1231.82	carbox.					[30,33]	[29]	
AM1Ac	C_62_H_109_N_11_O_14_	1231.82	carbox. cycl.					[30]		
AM19	C_62_H_111_N_11_O_14_	1233.83	+OH			+OH		[31,33]	[29]	[26]
AM1c9	C_62_H_111_N_11_O_14_	1233.83	cycl.			+OH		[33]	[29]	
AM49	C_62_H_111_N_11_O_14_	1233.83		+OH		+OH		[31,33]		[26]
AM69	C_62_H_111_N_11_O_14_	1233.83			+OH	+OH		[31,33]		[26]
AM11d	C_62_H_111_N_11_O_14_	1233.83	+2×OH					[33]		
*UM19.8min*	C_61_H_109_N_11_O_15_	1235.81	+OH	-CH_3_			+2×OH	[33]		
*UM23.0min*	C_61_H_109_N_11_O_15_	1235.81		-CH_3_			+3×OH	[33]		
AM1DI9	C_62_H_113_N_11_O_14_	1235.85	+OH +sat.			+OH		[31]		
*UM26.0min*	C_62_H_109_N_11_O_15_	1247.81	carbox.				+OH	[33]		
*UM20.6min*	C_62_H_111_N_11_O_15_	1249.83	+OH				+2×OH	[33]		
*UM22.4min*	C_62_H_111_N_11_O_15_	1249.83	+OH				+2×OH	[33]		
*UM24.4min*	C_62_H_111_N_11_O_15_	1249.83	+OH				+2×OH	[33]		
*UM25.5min*	C_62_H_111_N_11_O_15_	1249.83	+OH				+2×OH	[33]		
AM1S	C_62_H_111_N_11_O_15_S	1281.80	+sul.					[32]	[32]	
AM1c-Glc	C_68_H_119_N_11_O_19_	1393.87	cycl. +glu.					[33]		

Abbreviations: ald. = aldehyde; carbox. = doubly oxygenated amino acid leading to a carboxyl group; cycl. = single oxygenation leading to a cyclized amino acid; glu. = glucuronic acid conjugate; sat. = saturation (reduction of carbon-carbon double bond); sul. = sulfate conjugate; UM = unknown metabolite. Substance codes presented in italics reflect previously unreported phase I metabolites for which exact positions of the added hydroxyl moieties were not provided.

### 3.2. Sample Analysis

The studied urine samples were obtained from the TransplantLines Biobank and Cohort Study for kidney (KTR) and (potential) liver transplant recipients (LTR) and were initially treated as four different substudies. Samples from study A were analyzed between 24 November and 3 December 2021, and this study included 570 KTR who had a functional graft for at least one year post-transplantation when samples were taken. Study B’s samples were analyzed between 8 and 17 December 2021, and this study included samples of 163 KTR at 3, 12, and 24 months after transplantation. Samples from study C were analyzed between 18 and 22 November 2021, and this study included 316 LTR who had a functional graft for at least one year post-transplantation when samples were taken. Study D’s samples were analyzed between 11 and 15 November 2021, and this study included samples of 176 (potential) LTR before and/or at various timepoints after transplantation (see Table S5). Analytical performance was ensured following assessment of the reproducibility of signal intensity values (see Figures S2–S5) and retention time stability of stable-isotope-labeled standards (see Figures S6–S9). In addition, the expected clustering of samples (based on immunosuppressive drug use [20]) in principal component analysis (PCA) was verified (see Figures S10–S13).

### 3.3. Characteristics of Kidney and Liver Transplant Recipients

For this specific study on the real-world metabolism of CsA, enlarged cohorts were constructed for KTR and LTR, respectively, by combining study A with the 24-month post-transplantation samples of study B and study C with the 12-month post-transplantation samples of study D. This combination resulted in enlarged cohorts of 732 KTR and 350 LTR, and corresponding PCA plots of pharmacometabolomics data reassuringly do not show separation between participants from the respective substudies (see Figures S15 and S16). A detailed overview of subject characteristics is provided in Table 2. Regarding these characteristics, CsA use was confirmed using PMx in 126 out of 732 KTR (17%) and 38 out of 350 LTR (11%). Of these 126 KTR, the median age was 59 (interquartile range [IQR] 51–66) years, and 49% were female. Of the 38 LTR, the median age was 60 (IQR 47–66) years, and 47% were female. In KTR, the kidney function marker estimated glomerular filtration rate (eGFR), serum albumin, and the liver function marker alanine aminotransferase (ALT) were significantly lower in CsA users than in nonusers. Time since transplantation was significantly longer in KTR who used CsA compared to KTR not using CsA. Furthermore, the use of the immunosuppressive drugs tacrolimus and mycophenolate mofetil (MMF) and the use of calcium blockers were significantly lower in CsA users than in nonusers. In LTR, the time since transplantation was significantly longer for CsA users than nonusers. Tacrolimus use and MMF use were significantly lower in LTR using CsA compared to those not using CsA, whereas prednisolone use is significantly higher in CsA users compared to nonusers.

### 3.4. Feature Selection

**Figure 2:**
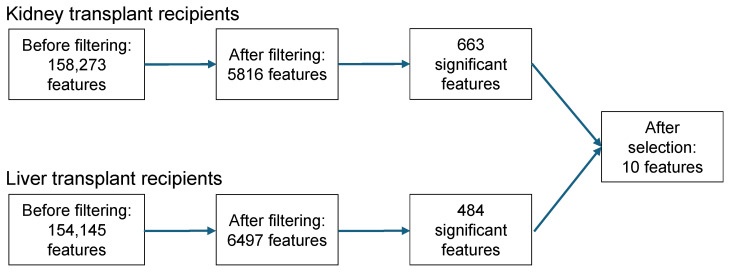
Schematic overview of feature selection in kidney and liver transplant recipients. Features were filtered based on *m*/*z* value (≥550) and retention time (≥14.0 min and ≤15.5 min). After filtering, a Mann–Whitney U test was performed, and corresponding *p*-values were Bonferroni-corrected. A *p*-value < 0.05 was considered statistically significant. Finally, significant features were assessed manually to exclude isotope peaks, adducts, and low abundance features.

Starting with 158,273 features in KTR, 663 were significantly associated with CsA use. In LTR, 484 features out of 154,145 features were significantly associated with CsA use. Of these features, the isotope peaks, adduct signals, in-source fragments, and low-abundance features were removed (manually). This feature removal resulted in a total of ten prioritized features, with nine features found in KTR and eight in LTR, of which seven overlapped (Table 3). See Figure 2 for a schematic overview of the entire selection process.

### 3.5. Metabolite Identification (As Level 3 “Putatively Characterized Compound Classes”, According to the Metabolomics Standards Initiative, MSI [22])

A manual assessment of cyclosporine-positive samples using the *m*/*z* values and retention times of the ten prioritized features (see Table 3) revealed thirteen distinct signals associated with cyclosporine exposure (see Table 4 and Figures S17–S26). One signal corresponded to cyclosporine itself and had a relative median metabolite abundance of 1.01% and 0.73% in KTR and LTR, respectively.

The major phase I metabolites of cyclosporine we expected to find in urine based on our literature study, demethylcyclosporine (AM4N) and hydroxycyclosporine (AM1), were identified based on the expected mass differences and lower retention times compared to CsA. For demethylcyclosporine, the median metabolite abundances were 1.41% and 1.38% in KTR and LTR, respectively. The median metabolite abundances for hydroxycyclosporine were 64.01% in KTR and 60.66% in LTR, making hydroxycylosporine the most abundant substance in both cohorts. It should, however, be noted that only one (rather broad) peak reflecting monohydroxylated CsA was detected, which possibly also captured the lower abundance CsA metabolites AM1c and AM9. The latter likely also applies to the five doubly oxygenated CsA metabolites (AM19, AM1c9, AM49, AM69, AM11d) for which only two signals were found, measuring the second and third highest median metabolite abundances (RT 14.2 min: 20.29% for KTR and 21.99% for LTR; RT 14.7 min: 4.35% for KTR and 4.75% for LTR). For the remaining expected urinary metabolites, signals corresponding to demethyldihydroxycyclosporine (AM4N69) and demethylhydroxycyclosporine (AM4N9) were found with median metabolite abundances below 3%, whereas cyclic hydroxycyclosporine (AM1c) and saturated hydroxycyclosporine (AM1DI) were not found, possibly not reaching the minimal abundance threshold.

Furthermore, we detected three additional metabolites in the urine of KTR and LTR that had not been detected in urine previously. Among these were the cyclosporine aldehyde (AM1AL) and a carboxylated CsA variant that have been detected in blood and/or bile before [29,30,33]. Interestingly, only one aldehyde variant has been reported before, while we found two distinct signals with median abundances around 1%. Conversely, two carboxylated variants (AM1A, AM1Ac) have previously been reported in blood and/or bile [29,30,33], and we only found one distinct signal. The report on AM1Ac in bile [33] may, however, have listed a wrong *m*/*z* value for their carboxylated and cyclized metabolite, which would logically match the low abundance hydroxycyclosporine carboxylic acid metabolite that we found.

In addition to the second CsA aldehyde variant, we found another unreported substance with an *m*/*z* value of 670.434. We did not assign an identity to this substance, yet its mass difference compared to CsA (+137.011) matches the mass of hydroxycysteine, thus potentially suggesting the involvement of glutathione conjugation. We were also unable to detect some metabolites previously only found in blood and/or bile, which could be due to their absence in urine but also due to the abundance threshold we employed. In this regard, cyclosporine glucuronide and cyclosporine sulfate could be detected in the urine of both KTR and LTR; yet, these metabolites did not pass the median abundance threshold and were, therefore, excluded from our analyses. These findings do, however, underscore the identification potential of the presented PMx workflow, demonstrating that even for a widely studied drug like CsA, multiple unreported metabolites could be detected.

Lastly, a notable strength of this study is the use of urinary PMx data of a large number of real-world cyclosporine users, allowing for insights into characteristics that may influence the variability in drug metabolism, particularly demographic, dietary, genetic, lifestyle, and physiological factors. However, this real-world setting also introduces a limitation, as these data inevitably encompass variability that remains unidentified and, therefore, cannot be explored in detail. Furthermore, participants included in our study come from a rather confined geographical area and, accordingly, may not be representative of populations in other regions. In addition, the biobank samples and untargeted analytical techniques utilized in our study (operated in a nonregulatory environment and without regulatory-grade method validation) may introduce certain (pre)analytical biases or complicate metabolite identification. The use of the SWATH data-independent acquisition mode, for example, generally produces more complex composite spectra compared to other acquisition modes and prompted us to generate cleaner product ion scan spectra for signals of interest as well. Moreover, the median abundance threshold of 1.0% relative to the highest measured median abundance as a cut-off value may be arbitrary, as some substances were, therefore, excluded from further analyses. Additionally, the detected metabolite patterns may not fully represent those at the time of urine excretion, as is the main trade-off associated with the wealth of information provided by 24-h urine sampling. Importantly, it was not our primary aim to completely characterize novel metabolites, and we acknowledge that such efforts would require the use of complementary techniques like nuclear magnetic resonance (NMR) and more comprehensive data analysis. Finally, current PMx research faces several challenges, including the need for more transparency, improved control of (pre)analytical factors, (more) validated protocols, larger sample sizes, accessible and reliable software, and enhanced analytical sensitivity to improve the accuracy and reproducibility of findings [11,13,34,35,36]. Variability in sample collection methods and metabolite identification strategies further complicate data interpretation [12,13,34,37,38,39,40]. Future efforts should focus on advanced data analysis and integration, for example, combining machine learning algorithms with innovative metabolomics methods, yielding more robust metabolomics findings and facilitating the translation of findings into clinical practice [12,13,37,41].

## 4. Conclusions

This study using cyclosporine as a model demonstrates how pharmacometabolomics can support real-world drug metabolism research. This study also highlights the complementary role of pharmacometabolomics in providing a real-world and phenotypic perspective on drug metabolism, which can enrich pharmacogenomics research and the corresponding clinical practices. Although the metabolism of cyclosporine has been extensively studied, our analysis revealed multiple metabolites previously undetected in urine, including metabolites that were previously identified in blood and/or bile, as well as “novel” metabolites (as level 3 “putatively characterized compound classes”, according to the Metabolomics Standards Initiative, MSI [22]). The observed metabolic patterns also allowed for the comparison of drug metabolism across different patient populations, revealing no notable differences between relatively large cohorts of kidney and liver transplant recipients. Assessing such profiles in other populations, for example, with varying geographic origins, genetic makeup, and comedication, could expand the phenotypic view of drug metabolism we provided. Hence, more studies are warranted to assess more patient heterogeneity and determine the clinical relevance of the novel metabolites and the metabolite profiles in terms of drug safety and effectiveness, ultimately leading to more personalized medicine.

## Figures and Tables

**Table 2:** Characteristics of 732 kidney transplant recipients and 350 liver transplant recipients.

	Kidney Transplant Recipients			Liver Transplant Recipients		
Characteristic As Median (IQR) or *n* (%)	CsA Users *n* = 126	CsA Nonusers *n* = 606	*p*-Value	CsA Users *n* = 38	CsA Nonusers *n* = 312	*p*-Value
Age (years)	59 (51, 66)	58 (48, 66)	**0.09**	60 (47, 66)	58 (46, 66)	0.95
Female sex	62 (49%)	230 (38%)	**0.03**	18 (47%)	134 (43%)	0.73
BMI (kg/m^2^)	26.7 (24.0, 29.6)	26.5 (23.9, 30.1)	0.68	25.7 (22.5, 27.5)	25.9 (23.3, 29.6)	0.34
Smoking status			0.92			0.27
Current	11 (11%)	48 (12%)		1 (3.3%)	31 (12%)	
Former	38 (39%)	147 (37%)		12 (40%)	79 (30%)	
Never	48 (49%)	202 (51%)		17 (57%)	154 (58%)	
Alcohol units per week	0.5 (0.0, 3.5)	1.2 (0.0, 6.2)	0.13	0.0 (0.0, 0.9)	0.0 (0.0, 0.5)	0.20
eGFR (mL/min/1.73 m^2^)	48 (37, 62)	55 (41, 66)	**0.009**	75 (61, 97)	73 (57, 92)	0.37
Serum albumin (g/L)	43 (41, 45)	44 (42, 46)	**0.02**	44 (42, 47)	44 (42, 46)	0.83
ALT (U/L)	17 (13, 22)	19 (14, 24)	**0.03**	26 (18, 31)	25 (18, 35)	0.96
Serum CRP (mg/L)	1.8 (0.7, 5.0)	1.8 (0.8, 4.6)	0.88	1.8 (0.7, 4.2)	2.0 (0.9, 4.7)	0.41
Serum glucose (mmol/L)	5.6 (5.1, 6.5)	5.5 (5.0, 6.3)	0.28	5.6 (5.0, 6.4)	5.6 (5.2, 6.7)	0.80
Urinary albumin (mg/24 h)	41 (11, 179)	32 (11, 147)	0.50	14 (9, 53)	13 (7, 49)	0.41
Time since transplantation (years)	9 (6, 17)	5 (2, 11)	**<0.001**	14 (10, 22)	8 (3, 17)	**<0.001**
Tacrolimus use	0 (0%)	451 (74%)	**<0.001**	2 (5.3%)	209 (67%)	**<0.001**
Mycophenolate use	75 (60%)	463 (76%)	**<0.001**	4 (11%)	86 (28%)	**0.04**
Azathioprine use	16 (13%)	65 (11%)	0.63	15 (39%)	74 (24%)	0.06
mTOR inhibitor use	1 (0.8%)	23 (3.8%)	0.15	1 (2.6%)	48 (15%)	0.06
Prednisolone use	125 (99%)	577 (95%)	0.07	23 (61%)	104 (33%)	**0.002**
Histamine H2-receptor antagonist use	3 (2.4%)	25 (4.1%)	0.50	1 (2.6%)	26 (8.3%)	0.36
Calcium channel blocker use	29 (23%)	238 (39%)	**<0.001**	8 (21%)	53 (17%)	0.69
ACE inhibitor use	40 (32%)	151 (25%)	0.14	3 (7.9%)	55 (18%)	0.20
Statin use	71 (56%)	336 (55%)	0.93	7 (18%)	70 (22%)	0.72

Abbreviations: CsA = cyclosporine A; BMI = body mass index; eGFR = estimated glomerular filtration rate; ALT = alanine aminotransferase; CRP = C-reactive protein; mTOR = mammalian target of rapamycin; ACE = angiotensin-converting enzyme. Characteristics are presented as median (interquartile range [IQR]) for continuous data and *n* (%) for categorical data. The Mann–Whitney U rank sum test was used for non-parametric, continuous variables, and the Chi-square test was applied to categorical variables. *p*-values in bold are statistically significant. In the kidney transplant recipient group, data on smoking were missing in 238 patients (32.5%); data on alcohol consumption were missing in 94 patients (12.8%); data on serum glucose were missing in 42 patients (5.7%), and data on urinary albumin were missing in 80 patients (10.9%). All other variables had missing data for <15 patients. In the liver transplant recipient group, data on smoking were missing in 56 patients (16%); data on alcohol consumption were missing in 34 patients (9.7%); data on serum glucose were missing in 19 patients (5.4%), and data on urinary albumin were missing in 41 patients (11.7%). All other variables had missing data for <3 patients.

**Table 3:** Overview of selected features.

		Kidney Transplant Recipients		Liver Transplant Recipients	
*m/z* ^1^	RT (min)	Rel. Median (%) ^2^	*p*-Value	Rel. Median (%) ^2^	*p*-Value
594.92	15.0	2.2	4.3 × 10^−152^	2.6	4.2 × 10^−63^
601.92	15.2	1.9	1.4 × 10^−150^	2.0	3.3 × 10^−60^
602.92	14.6	4.0	1.1 × 10^−150^	3.9	3.9 × 10^−48^
608.92	14.6	3.4	2.8 × 10^−138^	-	n.s.
608.92	14.8	-	n.s.	3.2	4.1 × 10^−66^
609.92	14.8	100.0	6.5 × 10^−142^	100.0	2.7 × 10^−40^
616.92	14.6	2.0	3.9 × 10^−154^	3.2	1.4 × 10^−67^
617.92	14.2	30.3	1.1 × 10^−150^	34.5	3.5 × 10^−66^
624.91	14.2	1.0	9.8 × 10^−152^	-	n.s.
670.43	14.1	1.4	3.9 × 10^−154^	2.0	2.9 × 10^−72^

Abbreviations: *m/z* = mass-to-charge ratio; n.s. = not significant; RT = retention time; rel. = relative. ^1^ CsA and its metabolites were expected as doubly charged ions [M+2H]^2+^. ^2^ For both the KTR and the LTR, the highest observed median intensity value of CsA users was set at 100%, and all other median values were expressed relative to the highest value.

**Table 4:** Overview of (putatively) identified cyclosporine metabolites (as level 3 “putatively characterized compound classes”, according to the Metabolomics Standards Initiative, MSI [22]).

Substance	Molecular Formula	Monoisotopic Mass	*m/z* ^1^	RT (min)	Median Metabolite Abundance ^2^ in KTR (%)	Median Metabolite Abundance ^2^ in LTR (%)
Demethylcyclosporine	C_61_H_109_N_11_O_12_	1187.83	594.92	15.0	1.41	1.38
Cyclosporine	C_62_H_111_N_11_O_12_	1201.84	601.92	15.2	1.01	0.73
Demethylhydroxycyclosporine	C_61_H_109_N_11_O_13_	1203.82	602.92	14.6 14.8	2.37 1.36	2.52 0.98
Cyclosporine aldehyde	C_62_H_109_N_11_O_13_	1215.82	608.92	14.6 14.8	0.75 1.27	0.73 1.25
Hydroxycyclosporine	C_62_H_111_N_11_O_13_	1217.84	609.92	14.8	64.01	60.66
Demethyldihydroxycyclosporine	C_62_H_113_N_11_O_13_	1219.85	610.92	14.3	1.10	1.19
Cyclosporine carboxylic acid	C_62_H_109_N_11_O_14_	1231.82	616.92	14.6	0.61	1.15
Dihydroxycyclosporine	C_62_H_111_N_11_O_14_	1233.83	617.92	14.2 14.7	20.29 4.35	21.99 4.75
Hydroxycyclosporine carboxylic acid	C_62_H_109_N_11_O_15_	1247.81	624.91	14.2	0.76	1.54
Unknown metabolite	C_65_H_118_N_12_O_15_S	1338.86	670.43	14.1	0.71	1.13

Abbreviations: *m*/*z* = mass-to-charge ratio; RT = retention time; KTR = kidney transplant recipients; LTR = liver transplant recipients. ^1^ CsA and its metabolites were expected and detected as doubly charged ions [M+2H]^2+^. ^2^ Median metabolite abundance values presented in the table reflect the median values (per study) of the relative quantitative readouts that were calculated by dividing the signal intensity of each substance individually by the sum of signal intensities of all substances found per cyclosporine user.

## Data Availability

The metabolomics datasets used in this study can be found at https://doi.org/10.26037/yareta:64ruex2sxff5nenyfyexurzs3m (accessed on 10 November 2024) (as substudies 2, 3, 4, and 5).

## Supplementary Materials

The following supporting information can be downloaded at www.mdpi.com/article/10.3390/metabo15010039/s1, Method S1: PubMed search strategy; Method S2: Preparation, use, and evaluation of intra-lab, long-term QC samples; Table S1: Overview of internal standards; Table S2: Overview of LC–MS analytical parameters; Table S3: Overview of MarkerView data (pre)processing settings; Table S4: Overview of PeakView chemical identification settings; Table S5: Overview of sample combinations for study D; Figure S1: PRISMA flow chart of study inclusion; Figure S2: Nonnormalized MS1-level absolute feature areas of the included internal standards, as were detected in the intra-lab, long-term QC samples included in study A; Figure S3: Nonnormalized MS1-level absolute feature areas of the included internal standards, as were detected in the intra-lab, long-term QC samples included in study B; Figure S4: Nonnormalized MS1-level absolute feature areas of the included internal standards, as were detected in the intra-lab, long-term QC samples included in study C; Figure S5: Nonnormalized MS1-level absolute feature areas of the included internal standards, as were detected in the intra-lab, long-term QC samples included in study D; Figure S6: Retention times of the MS1-level signals extracted for the included internal standards, as were detected in the intra-lab, long-term QC samples included in study A; Figure S7: Retention times of the MS1-level signals extracted for the included internal standards, as were detected in the intra-lab, long-term QC samples included in study B; Figure S8: Retention times of the MS1-level signals extracted for the included internal standards, as were detected in the intra-lab, long-term QC samples included in study C; Figure S9: Retention times of the MS1-level signals extracted for the included internal standards, as were detected in the intra-lab, long-term QC samples included in study D; Figure S10: Pareto-scaled scores plots for unsupervised principal component analysis of unnormalized MS1-level feature data of study A; Figure S11: Pareto-scaled scores plots for unsupervised principal component analysis of unnormalized MS1-level feature data of study B; Figure S12: Pareto-scaled scores plots for unsupervised principal component analysis of unnormalized MS1-level feature data of study C; Figure S13: Pareto-scaled scores plots for unsupervised principal component analysis of unnormalized MS1-level feature data of study D; Figure S14: Exemplary spectral library matching-based identification of hydroxylated cyclosporine A, as was found in urine of a kidney transplant recipient; Figure S15: Pareto-scaled scores and loadings plots for unsupervised principal component analysis of unnormalized MS1-level feature data of all samples of study A and the 24 months post-transplantation samples of study B; Figure S16: Pareto-scaled scores and loadings plots for unsupervised principal component analysis of unnormalized MS1-level feature data of all samples of study C and the 24 months post-transplantation samples of study D. Figures S17–S26: Exemplary extracted ion chromatograms and fragment spectra of cyclosporine A (as “identified compound”) and putative cyclosporine A metabolites (as “putatively characterized compound classes”).
