# Supplementary material for: Pharmacometabolomics Enables Real-World Drug Metabolism Sciences
Source: Metabolites. 2025 Jan 10;15(1):39. doi: 10.3390/metabo15010039 (PMC11767479; doi:10.3390/metabo15010039)
Supplement: Supplementary file 1 [file metabolites-15-00039-s001.zip › metabolites-3371789-supplementary.pdf]

Supplementary Material

Pharmacometabolomics enables real-world drug metabolism sciences

Fleur B. Nijdam[1], Marieke A.J. Hof[2], Hans Blokzijl[3], Stephan J.L. Bakker[4], Eelko Hak[1], Gérard Hopfgartner[5], Frank Klont[1,6]

[1]*Unit of PharmacoTherapy, -Epidemiology, and -Economics, University of Groningen, Groningen, The Netherlands*

[2]*Department of Analytical Biochemistry, University of Groningen, Groningen, The Netherlands*

[3]*Department of Gastroenterology, University Medical Center Groningen, University of Groningen, Groningen, The Netherlands*

[4]*Division of Nephrology, Department of Internal Medicine, University Medical Center Groningen, University of Groningen, Groningen, The Netherlands*

[5]*Life Sciences Mass Spectrometry, Department of Inorganic and Analytical Chemistry, University of Geneva, Genève, Switzerland*

[6]*Department of Clinical Pharmacy and Pharmacology, University Medical Center Groningen, University of Groningen, Groningen, The Netherlands*

Corresponding author: Dr. Frank Klont, Antonius Deusinglaan 1, 9713 AV Groningen, The Netherlands. Tel: +316-31921379. E-mail: frank.klont@rug.nl. ORCID: 0000-0003-3503-1694.

Supplementary Appendix

**Table of Contents**	**Page**
Supplementary Method S1: PubMed search strategy.	5
Supplementary Method S2: Preparation, use, and evaluation of intra-lab, long-term QC samples.	6
Supplementary Table S1: Overview of internal standards.	8
Supplementary Table S2: Overview of LC-MS analytical parameters.	9
Supplementary Table S3: Overview of MarkerView data (pre)processing settings.	11
Supplementary Table S4: Overview of PeakView chemical identification settings.	12
Supplementary Table S5: Overview of sample combinations for study D.	13
Supplementary Figure S1: PRISMA flow chart of study inclusion.	14
Supplementary Figure S2: Nonnormalized MS1-level absolute feature areas of the included internal standards, as were detected in the intra-lab, long-term QC samples included in study A.	15
Supplementary Figure S3: Nonnormalized MS1-level absolute feature areas of the included internal standards, as were detected in the intra-lab, long-term QC samples included in study B.	16
Supplementary Figure S4: Nonnormalized MS1-level absolute feature areas of the included internal standards, as were detected in the intra-lab, long-term QC samples included in study C.	17
Supplementary Figure S5: Nonnormalized MS1-level absolute feature areas of the included internal standards, as were detected in the intra-lab, long-term QC samples included in study D.	18
Supplementary Figure S6: Retention times of the MS1-level signals extracted for the included internal standards, as were detected in the intra-lab, long-term QC samples included in study A.	19
Supplementary Figure S7: Retention times of the MS1-level signals extracted for the included internal standards, as were detected in the intra-lab, long-term QC samples included in study B.	20
Supplementary Figure S8: Retention times of the MS1-level signals extracted for the included internal standards, as were detected in the intra-lab, long-term QC samples included in study C.	21

Supplementary Figure S9: Retention times of the MS1-level signals extracted for the included internal standards, as were detected in the intra-lab, long-term QC samples included in study D.	23
Supplementary Figure S10: Pareto-scaled scores plots for unsupervised principal component analysis of nonnormalized MS1-level feature data of study A.	24
Supplementary Figure S11: Pareto-scaled scores plots for unsupervised principal component analysis of nonnormalized MS1-level feature data of study B.	25
Supplementary Figure S12: Pareto-scaled scores plots for unsupervised principal component analysis of nonnormalized MS1-level feature data of study C.	25
Supplementary Figure S13: Pareto-scaled scores plots for unsupervised principal component analysis of nonnormalized MS1-level feature data of study D.	26
Supplementary Figure S14: Exemplary spectral library matching-based identification of hydroxylated cyclosporine A, as was found in urine of a kidney transplant recipient.	27
Supplementary Figure S15: Pareto-scaled scores and loadings plots for unsupervised principal component analysis of nonnormalized MS1-level feature data of all samples of study A and the 24 months post-transplantation samples of study B.	28
Supplementary Figure S16: Pareto-scaled scores and loadings plots for unsupervised principal component analysis of nonnormalized MS1-level feature data of all samples of study C and the 24 months post-transplantation samples of study D.	29
Supplementary Figure S17: MS1-level extracted ion chromatogram, SWATH/MS fragment spectrum, and product ion scan fragment spectrum of a putatively demethylated version of cyclosporine A (-14 Da) observed in urine of a human cyclosporine A user.	30
Supplementary Figure S18: MS1-level extracted ion chromatogram, SWATH/MS fragment spectrum, and product ion scan fragment spectrum of cyclosporine A observed in urine of a human cyclosporine A user.	31
Supplementary Figure S19: MS1-level extracted ion chromatogram, SWATH/MS fragment spectra, and product ion scan fragment spectra of two putatively demethylated and oxygenated versions of cyclosporine A (-14, +16 Da) observed in urine of a human cyclosporine A user.	32
Supplementary Figure S20: MS1-level extracted ion chromatogram, SWATH/MS fragment spectra, and product ion scan fragment spectra of two putatively cyclized, oxygenated, and dehydrogenated versions of cyclosporine A (-2/+2, +16, -2 Da) observed in urine of a human cyclosporine A user.	34

Supplementary Figure S21: MS1-level extracted ion chromatogram, SWATH/MS fragment spectrum, and product ion scan fragment spectrum of a putatively oxygenated version of cyclosporine A (+16 Da) observed in urine of a human cyclosporine A user.	36
Supplementary Figure S22: MS1-level extracted ion chromatogram, SWATH/MS fragment spectrum, and product ion scan fragment spectrum of a putatively demethylated and doubly oxygenated version of cyclosporine A (-14, +32 Da) observed in urine of a human cyclosporine A user.	37
Supplementary Figure S23: MS1-level extracted ion chromatogram, SWATH/MS fragment spectrum, and product ion scan fragment spectrum of a putatively carboxylated version of cyclosporine A (+32/-2 Da) observed in urine of a human cyclosporine A user.	38
Supplementary Figure S24: MS1-level extracted ion chromatogram, SWATH/MS fragment spectra, and product ion scan fragment spectra of two putatively doubly oxygenated versions of cyclosporine A (+32 Da) observed in urine of a human cyclosporine A user.	39
Supplementary Figure S25: MS1-level extracted ion chromatogram, SWATH/MS fragment spectrum, and product ion scan fragment spectrum of a putatively oxygenated and carboxylated version of cyclosporine A (+16, +32/-2 Da) observed in urine of a human cyclosporine A user.	41
Supplementary Figure S26: MS1-level extracted ion chromatogram, SWATH/MS fragment spectrum, and product ion scan fragment spectrum of a putative product of oxygenation and glutathione conjugation of cyclosporine A observed in urine of a human cyclosporine A user.	42

Method S1. PubMed search strategy.

A literature search was conducted on PubMed using the search terms “cyclosporine”, “ciclosporine”, “CsA”, and “drug metabolism”, “metabolite”, and “human”, “man”, “men”, “volunteer”, “woman”, “women” in the title and/or abstract. The truncated form of the terms was used when applicable (“cyclosporin*”, “ciclosporin*”, “drug metabol*”, “human*”, “volunteer*”). The last search was performed on July 10th 2024, and no time restriction nor other filters were applied.

Method S2. Preparation, use, and evaluation of intra-lab, long-term QC samples.

First morning void urine of noncommercial sources was collected in 50 mL polypropylene tubes (Greiner Bio-One; Cat. No. 227261) and was kept at room temperature. Within 3 hours after collection, samples were mixed in a clean borosilicate glass Erlenmeyer flask by gentle swirling for approximately one minute. Next, the pooled urine was aliquoted in 1.5 mL polypropylene microcentrifuge tubes (Eppendorf; Cat. No. 0030125150) using non-sterile polypropylene tips, and the aliquots were stored at -25 °C until use, after which the remaining volume was discarded.

In general, QC samples were processed in the same manner as the study samples, and eight QC samples were included per analytical batch of sixty study samples. Two of them were analyzed at the start of the batch, and two were analyzed at the end of the batch, which were followed by a blank sample consisting of 100% water (of which the total ion current chromatogram was inspected visually in the SCIEX Analyst TF software (version 1.7.1) for the absence of sample related signals indicative of apparent carryover effects as could lead to discarding of the batch (which was not needed for any of the studies presented in this work) or to instigating corrective actions during data analysis and reporting, which is feature dependent and should be evaluated on a case by case basis). The other four QC samples were distributed evenly over the batch (and were thus analyzed after study sample 12, 24, 36, and 48). Regarding the first two QC samples, these were preceded by three conditioning urine samples that consisted of the same QC urine material but without the addition of internal standards. Then, the first QC samples were quickly screened on the flight with the SCIEX Analyst TF software (version 1.7.1) for signal intensities and retention times of the internal standards diclofenac-$^{13}C_6$ and caffeine-$^{13}C_3$ based on which it was decided to continue the batch (which was the case for all batches of the studies presented in this work). In general, no action was taken if signals were not deviating more than an order of magnitude from the expected value and when retention times were within half a minute of the expected value, as the corresponding variation could be accounted for through data normalization and retention time correction procedures, respectively. These criteria were also used when evaluating all the QC samples and were taking into account all five internal standards after completion of each analytical batch (see supplementary Figures S2 to S9 for the corresponding results of the presented studies). For these assessments, however, we did not use the aforementioned software, but we used a custom-modified version of the SCIEX MarkerView software (version 1.3.1) to extract nonnormalized MS1 feature intensity values (using the settings presented in supplementary Table S3) and SCIEX MultiQuant software (version 2.1) to extract the MS1-level retention times with a ±2.5 mDa mass extraction window and a 2.0-point Gaussian smoothing width.

Regarding the nonnormalized MS1 feature data, these data relied on the quality of the analysis but also on the performance of the peak picking algorithm employed. Hence, we accepted larger degrees of variation in within-batch responses of the internal standards measured in the QC samples

as compared to internal standards in QC samples measured by targeted assays and being processed in a selected reaction monitoring-type of manner. In general, we accepted batches when the coefficients of variations for all five internal standards were within 30%, for which we took into account non-outlier values given that zero values or abnormally high values could arise from analytical issues, peak picking issues, and a combination of both. We did take corrective actions in case we observed outlier values for all five internal standards measured in the same QC sample, which led to a rejection of all of the twelve preceding and twelve ensuing study samples (which was not needed for any of the studies presented in this work). When we furthermore observed outlier values for all five internal standards in three or more QC samples, the entire batch was rejected (which was not needed for any of the studies presented in this work).

Lastly, a check involving the QC samples was conducted based on principal component analysis of the aforementioned nonnormalized MS1 features using a custom-modified version of the SCIEX MarkerView software (version 1.3.1) with the settings presented in supplementary Table S3. For the corresponding data inspection, no clear-cut quality criteria were employed other than that the plots should indicate clustering of the QC samples (see supplementary Figures S10 to S13 for the corresponding results of the presented studies, which all showed clustering of the QC samples).

Table S1. Overview of internal standards.

Chemical	PubChem CID	Isotope label	Supplier	Catalogue number
Acetaminophen	1983	ring-D_4	Toronto Research Chemicals	A161222
Diclofenac	3033	ring-$^{13}C_6$	Sigma-Aldrich	35361
Naproxen	156391	methoxy-D_3	Sigma-Aldrich	32104
Caffeine	2519	trimethyl-$^{13}C_3$	Sigma-Aldrich	C-082
Cotinine	854019	N-methyl-D_3	Sigma-Aldrich	C-035

Table S2. Overview of LC-MS analytical parameters.

Setting	Value
Analytical setup:	
LC pump	Dionex Ultimate 3000 RS Binary Pump (Cat. No. HPG-3400RS)
LC autosampler	Dionex Ultimate 3000 TS Autosampler (Cat. No. WPS-3000TRS)
LC column oven	Dionex Ultimate 3000 RS Column Compartment (Cat. No. TCC-3000RS)
LC operation software	Dionex Chromeleon Software (version 6.80 SR10, Build 2818)
MS instrument	SCIEX TripleTOF® 5600 Mass Spectrometer (Cat. No. 1032150)
MS calibration device	SCIEX Calibrant Delivery System (Cat. No. ISG-002019)
MS operation software	SCIEX Analyst TF Software (version 1.7.1, Build 1163)
LC parameters:	
Autosampler temperature	6 °C
Column temperature	40 °C
Analytical column	Dr. Maisch ReproSil Gold 120, 2.5 µm, 120 Å, 2.0 × 150 mm (Cat. No. r125.9g.s1502)
Guard column	Dr. Maisch ReproSil Gold 120, 2.5 µm, 120 Å, 2.0 × 5 mm (Cat. No. r125.9g.v0002)
Mobile phase A	5 mM ammonium formate (Fluka; Cat. No. 17843) and 0.1% formic acid (Merck; Cat. No. 1.00264.0100) in water (Chemsolute; Cat. No. 470.2500)
Mobile phase B	Methanol (Fisher Science; Cat. No. M/4058/17)
Trapping setting	0.3 mL/min at 5% B
Flow rate	0.3 mL/min
LC program	0.20 min: column switch<br>0.50 min: 5% B<br>12.50 min: 80% B<br>14.00 min: 100% B<br>17.00 min: 100% B<br>17.01 min: 5% B<br>19.00 min: 5% B
MS parameters:	
Source temperature (TEM)	500 °C
Curtain gas (CUR)	35 psi
Nebulizer gas (GS1)	40 psi
Heater gas (GS2)	40 psi
IonSpray voltage floating (ISVF)	5,200 V
Declustering potential (DP)	80 V
Collision energy (CE) MS1	10 V
Collision energy (CE) MS2	40 V
Collision energy spread (CES) MS1	0 V
Collision energy spread (CES) MS2	30 V
Accumulation time MS1	50 ms
Accumulation time MS2	23 ms
TOF MS1 range	m/z 100-1,250
TOF MS2 range	m/z 40-1,250
SWATH precursor isolation windows	Experiment 1: m/z 100-115<br>Experiment 2: m/z 114-129<br>Experiment 3: m/z 128-143<br>Experiment 4: m/z 142-157<br>Experiment 5: m/z 156-171<br>Experiment 6: m/z 170-185<br>Experiment 7: m/z 184-199<br>Experiment 8: m/z 198-213<br>Experiment 9: m/z 212-227<br>Experiment 10: m/z 226-241<br>Experiment 11: m/z 240-255<br>Experiment 12: m/z 254-269<br>Experiment 13: m/z 268-283<br>Experiment 14: m/z 282-297<br>Experiment 15: m/z 296-311<br>Experiment 16: m/z 310-325<br>Experiment 17: m/z 324-339<br>Experiment 18: m/z 338-353<br>Experiment 19: m/z 352-367<br>Experiment 20: m/z 366-381<br>Experiment 21: m/z 380-395<br>Experiment 22: m/z 394-409<br>Experiment 23: m/z 408-423<br>Experiment 24: m/z 422-437<br>Experiment 25: m/z 436-451<br>Experiment 26: m/z 450-465<br>Experiment 27: m/z 464-479

	Experiment 28: m/z 478-493<br>Experiment 29: m/z 492-507<br>Experiment 30: m/z 506-521<br>Experiment 31: m/z 520-535<br>Experiment 32: m/z 534-549<br>Experiment 33: m/z 548-563<br>Experiment 34: m/z 562-577<br>Experiment 35: m/z 576-591<br>Experiment 36: m/z 590-605<br>Experiment 37: m/z 604-900<br>Experiment 38: m/z 899-1,250
Total cycle time	± 1.0 s
Mass calibration strategy	Manually before each batch and automatically every 3 samples (± once per hour)
Mass calibration compounds MS1	purine (PubChem CID 1044, m/z = 121.0509)<br>caffeine (PubChem CID 2519, m/z = 195.0877)<br>clomipramine (PubChem CID 2801, m/z = 315.1623)<br>verapamil (PubChem CID 2520, m/z = 455.2904)<br>reserpine (PubChem CID 5770, m/z = 609.2807)<br>hexakis(2,2,3,3-tetrafluoropropoxy)phosphazene (PubChem CID 2775076, m/z = 922.0098)<br>hexakis(1H,1H,5H-perfluoropentoxy)phosphazene (PubChem CID 2775070, m/z = 1521.9715)
Mass calibration compound MS2	reserpine (PubChem CID 5770, m/z = 609.2807)

Table S3. Overview of MarkerView data (pre)processing settings.

Setting	Value
Feature finding:	
Experiment	MS1
Minimum retention time	0.50 min
Maximum retention time	16.00 min
Subtraction offset	15 scans
Subtraction multiplication factor	1.3
Noise threshold	5
Minimum spectral peak width	5 ppm
Minimum retention time peak width	5 scans
Assign charge states	Enabled
Feature alignment:	
Retention time tolerance	0.50 min
Mass tolerance	20 ppm
Feature filtering:	
Maximum number of peaks	8,000,000*
Remove peaks in < N samples	Disabled
Isotope filtering	Disabled
Intensity threshold	5
Use exclusion list	Disabled
Retention time filtering	Disabled
Use area integrated from raw data, not from original peak finding	Disabled
Principle component analysis:	
PCA preprocessing - Weighting	None
PCA preprocessing - Scaling	Pareto
Perform PCA-DA (supervised)	Disabled

*: This parameter was set high enough to prevent peaks from getting filtered at this stage.

Table S4. Overview of PeakView chemical identification settings.

Setting	Value
Calculations:	
Do not calculate details for XIC with intensity < N counts or S:N < M	N = 1000, M = 100
Default XIC width (Da)	0.01
Default retention time width	0.2
Default threshold (cps)	100
Default threshold (ratio of control)	1
Non-targeted peak finding	Not selected
Formula Finder	Not selected
Library searching:	
Algorithm to use during library search	Candidate search
Results sorted by	Fit
Libraries to search	-SCIEX Forensics version 1.1<br>-Custom library built following the analysis of a commercial reference standard of cyclosporine A (Sigma-Aldrich, Cat. No. PHR1092)
Precursor mass tolerance	0.4 Da
Collision energy tolerance	Not selected
Retention time tolerance	Not selected
Mass tolerance	0.4 Da
Use polarity	Selected
Use collision energy spread	Not selected
Use compound specific purity	Not selected
Maximum number of hits	5
Intensity threshold	0.05
Minimal purity	10%
Intensity factor	5
Confidence settings:	
Mass error – hit	< 5 ppm
Mass error – potential hit	< 10 ppm
Mass error – contribution to combined score	40%
Isotope ratio – hit	< 10%
Isotope ratio – potential hit	< 20%
Isotope ratio – contribution to combined score	10%
Library score – hit	> 70
Library score – potential hit	> 30
Library score – contribution to combined score	50%
Combined score for a positive identification	≥ 80%

Table S5. Overview of sample combinations for study D.

Sample combinations	Number of instances
screening only	108
screening + 3 months	7
screening + 3 months + 6 months	6
screening + 3 months + 12 months	4
screening + 3 months + 6 months + 12 months	5
screening + 3 months + 6 months + 12 months + 24 months	4
screening + 6 months	7
screening + 6 months + 12 months	2
screening + 6 months + 24 months	1
screening + 6 months + 12 months + 24 months	1
screening + 12 months	3
screening + 12 months + 24 months	1
3 months only	4
3 months + 6 months	1
3 months + 24 months	1
3 months + 6 months + 12 months	1
6 months only	4
6 months + 24 months	2
6 months + 12 months + 24 months	2
12 months only	4
12 months + 24 months	7
24 months only	1

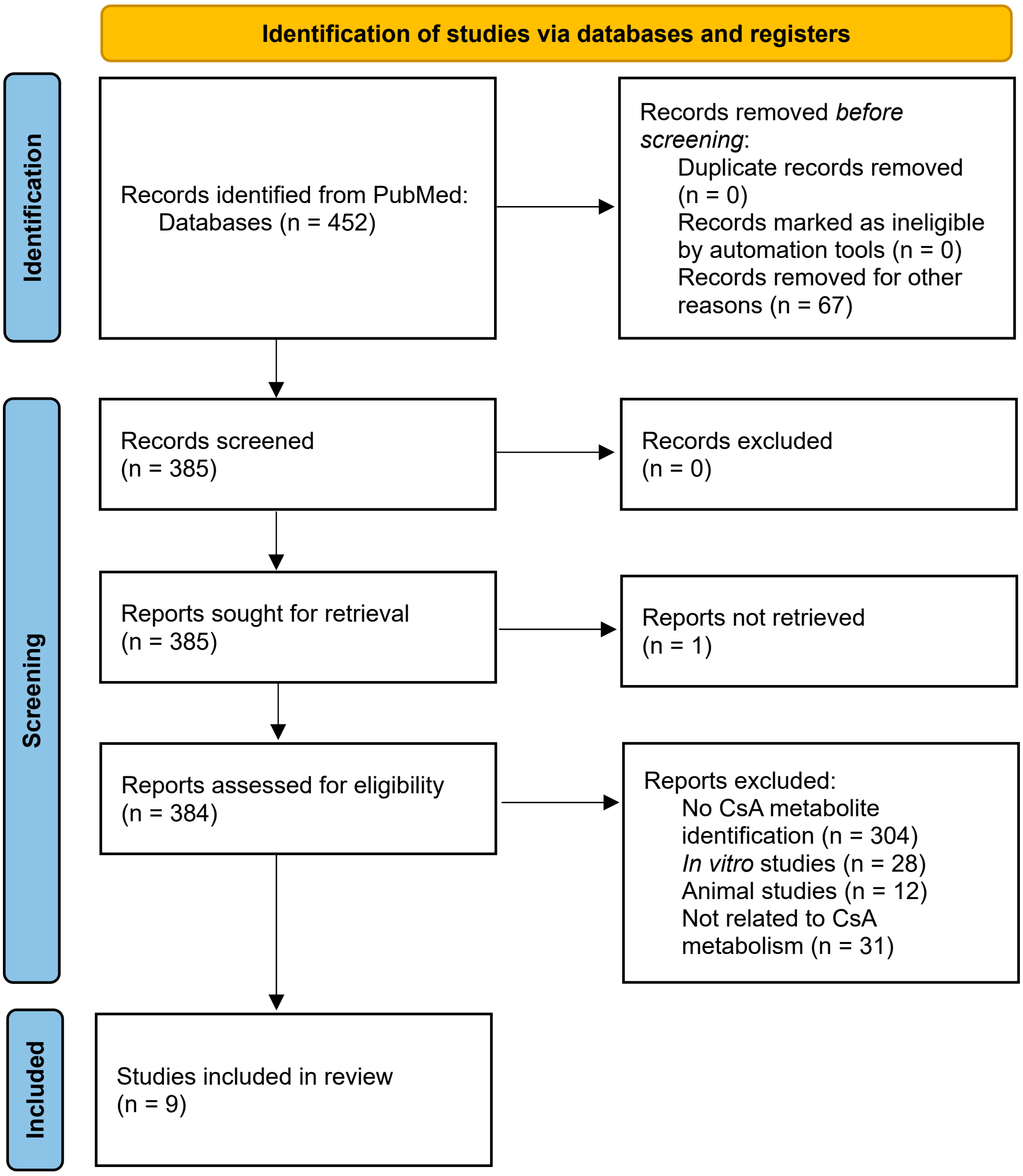

Figure S1. PRISMA flow chart of study inclusion. The 67 articles removed for other reasons included non-English articles (3), reviews (62), and case reports (2).

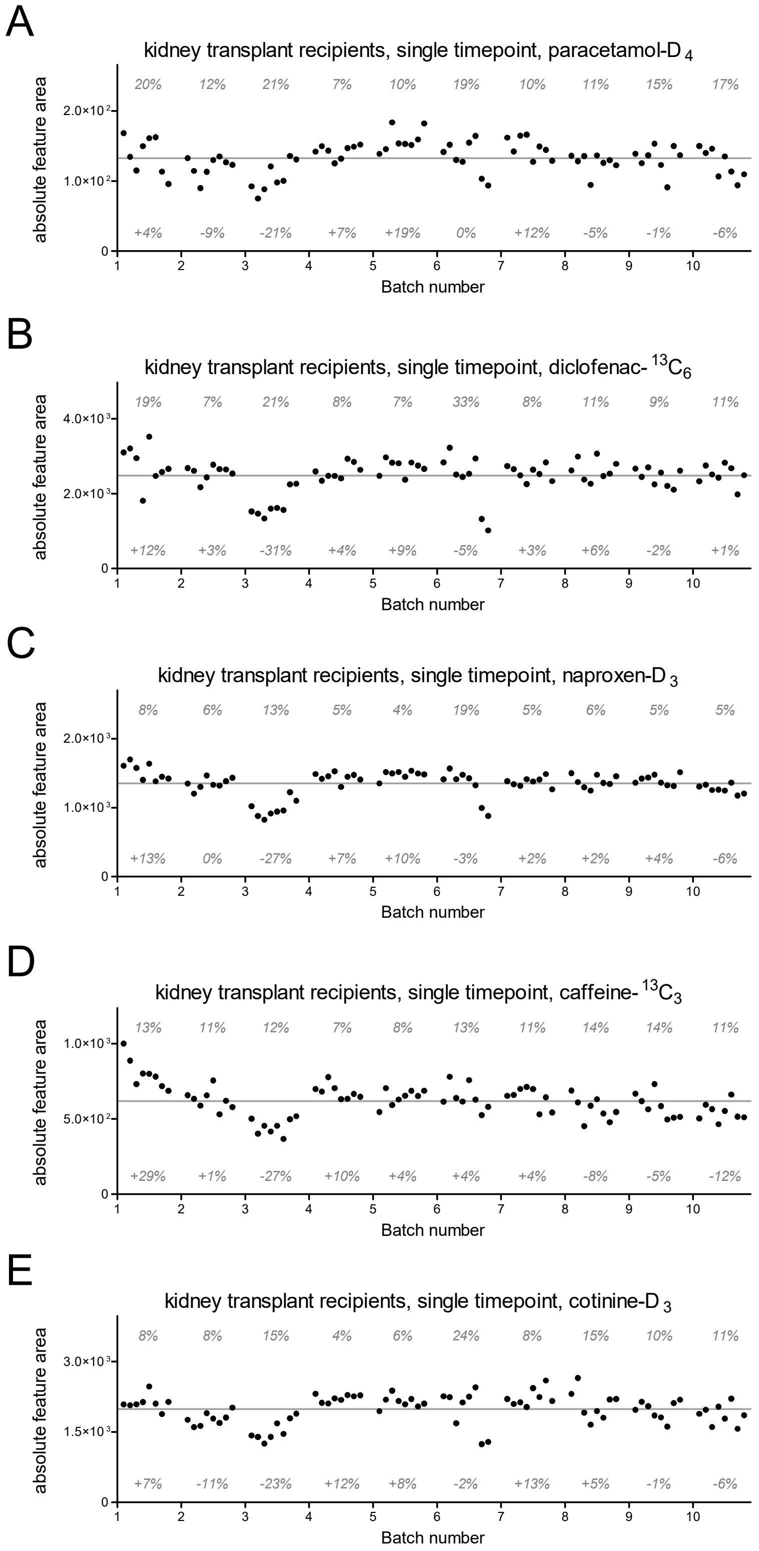

Figure S2. Nonnormalized MS1-level absolute feature areas of the included internal standards, as were detected in the intra-lab, long-term QC samples included in study A. The non-negative percentages presented in grey at the top of each subfigure represent coefficients of variations calculated per batch, and the percentages presented in grey at the bottom of each subfigure represent the biases of the average values measured per batch relative to the average values measured in the entire study.

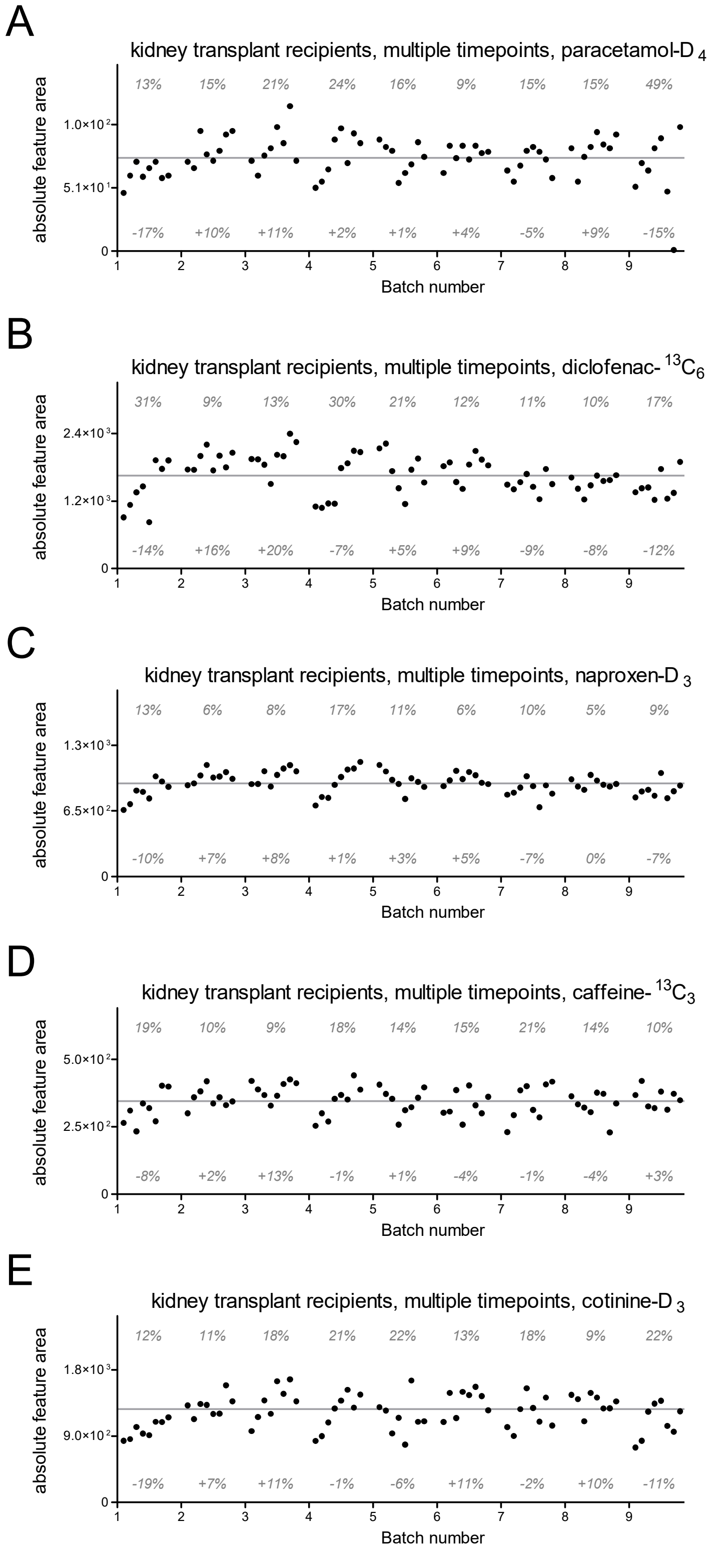

Figure S3. Nonnormalized MS1-level absolute feature areas of the included internal standards, as were detected in the intra-lab, long-term QC samples included in study B. The non-negative percentages presented in grey at the top of each subfigure represent coefficients of variations calculated per batch, and the percentages presented in grey at the bottom of each subfigure represent the biases of the average values measured per batch relative to the average values measured in the entire study.

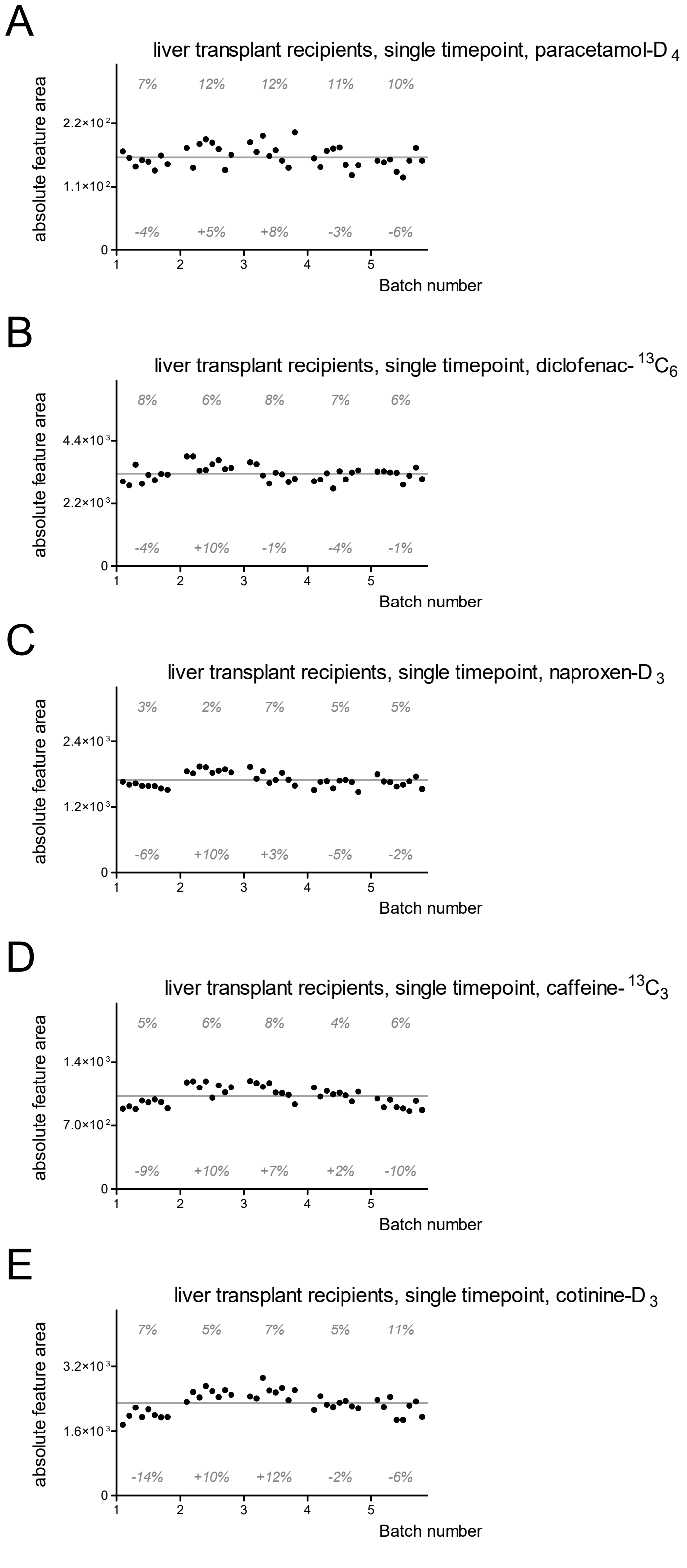

Figure S4. Nonnormalized MS1-level absolute feature areas of the included internal standards, as were detected in the intra-lab, long-term QC samples included in study C. The non-negative percentages presented in grey at the top of each subfigure represent coefficients of variations calculated per batch, and the percentages presented in grey at the bottom of each subfigure represent the biases of the average values measured per batch relative to the average values measured in the entire study.

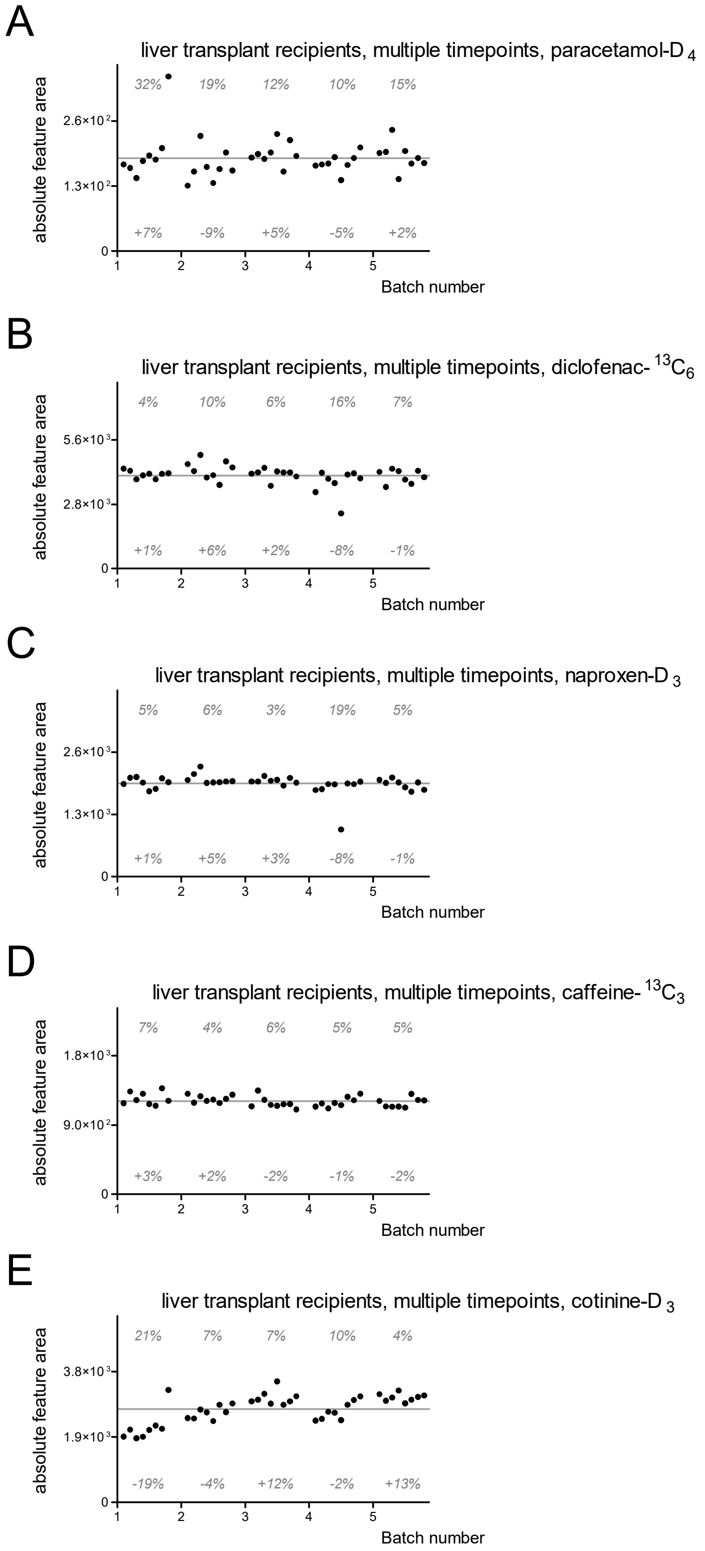

Figure S5. Nonnormalized MS1-level absolute feature areas of the included internal standards, as were detected in the intra-lab, long-term QC samples included in study D. The non-negative percentages presented in grey at the top of each subfigure represent coefficients of variations calculated per batch, and the percentages presented in grey at the bottom of each subfigure represent the biases of the average values measured per batch relative to the average values measured in the entire study.

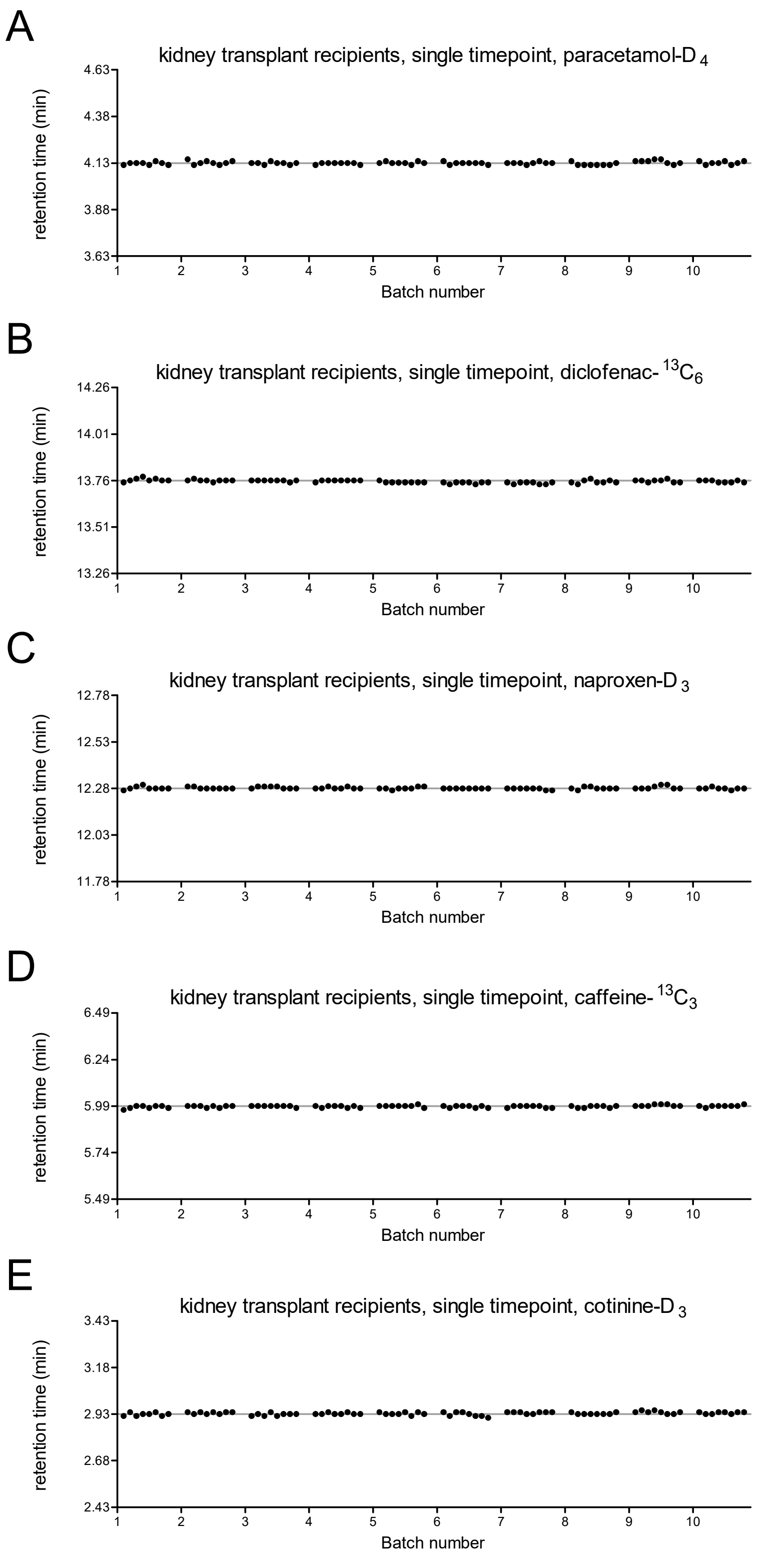

Figure S6. Retention times of the MS1-level signals extracted for the included internal standards, as were detected in the intra-lab, long-term QC samples included in study A.

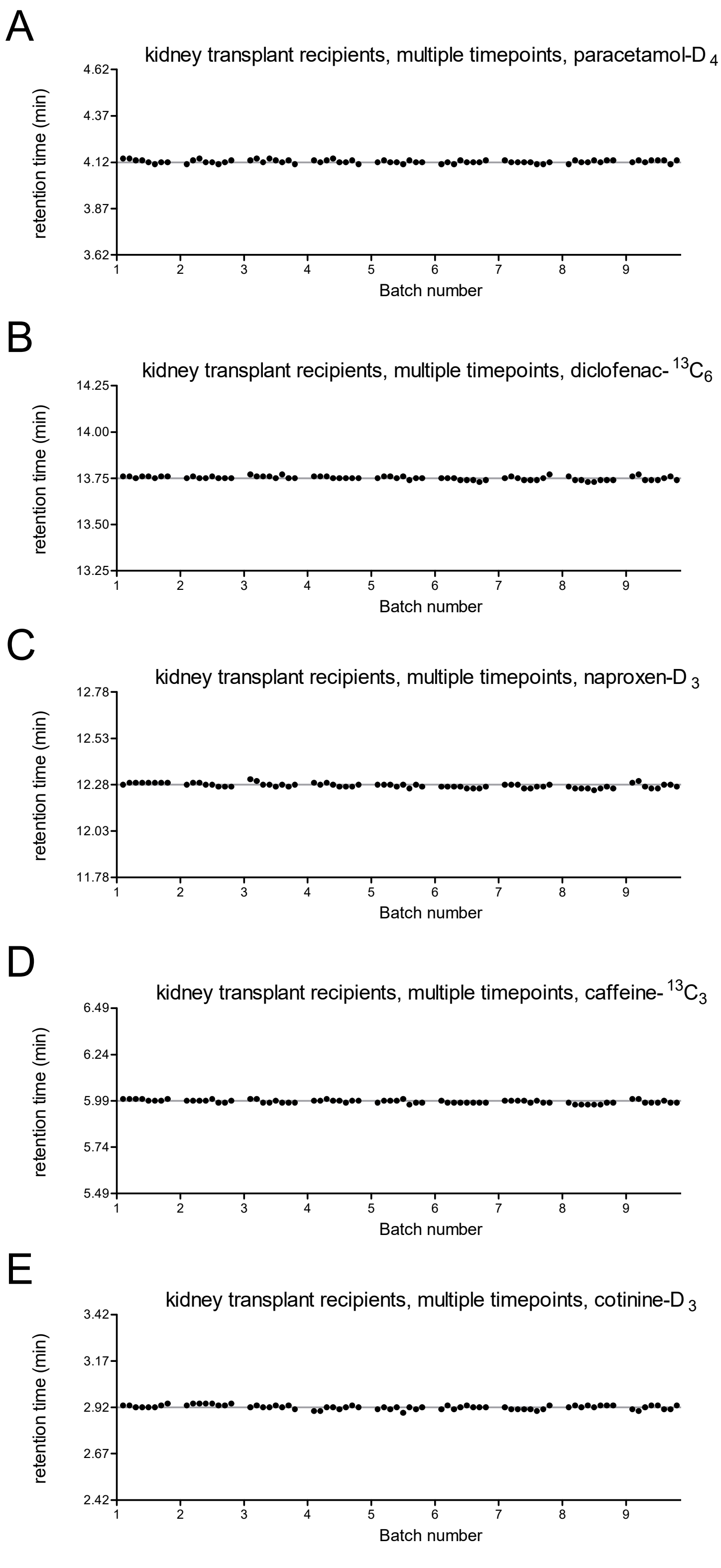

Figure S7. Retention times of the MS1-level signals extracted for the included internal standards, as were detected in the intra-lab, long-term QC samples included in study B.

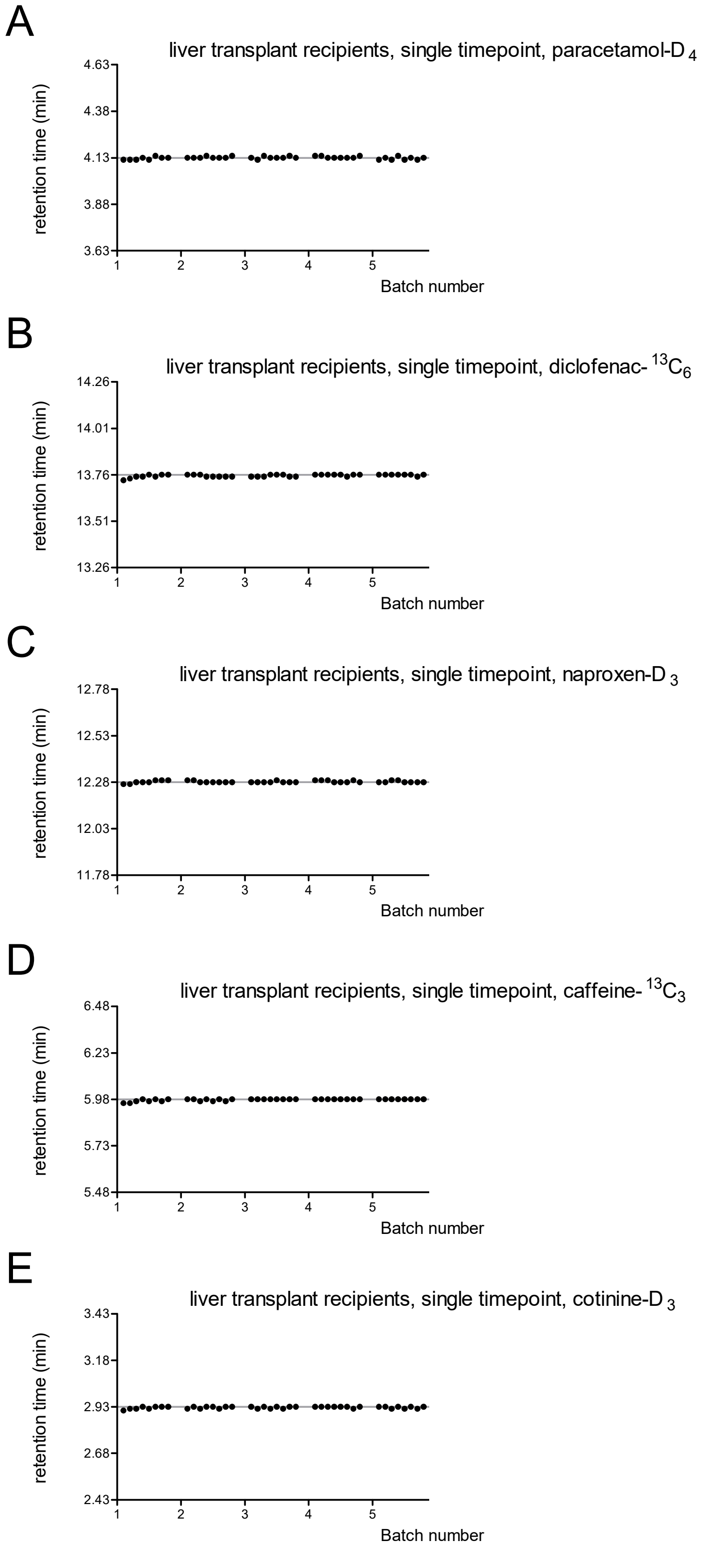

Figure S8. Retention times of the MS1-level signals extracted for the included internal standards, as were detected in the intra-lab, long-term QC samples included in study C.

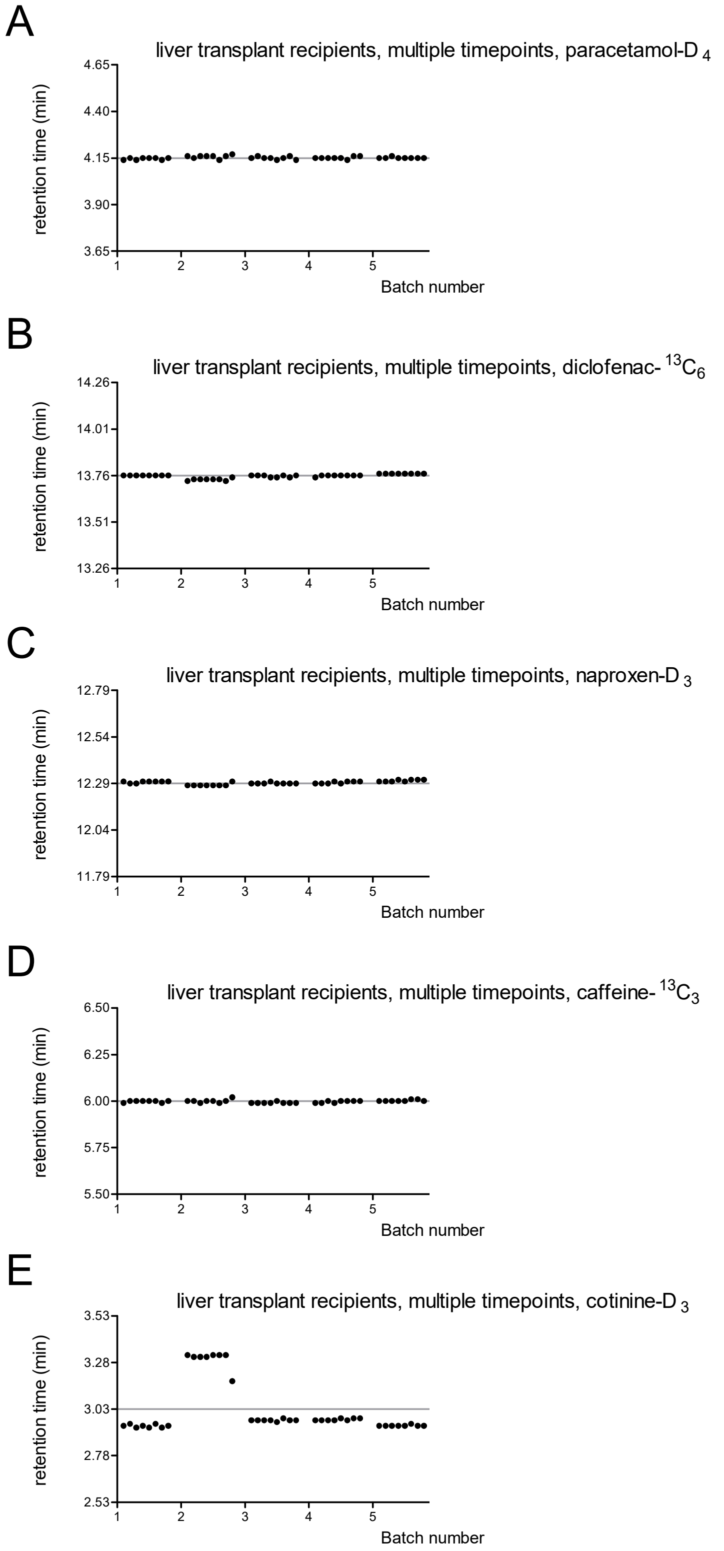

Figure S9. Retention times of the MS1-level signals extracted for the included internal standards, as were detected in the intra-lab, long-term QC samples included in study D.

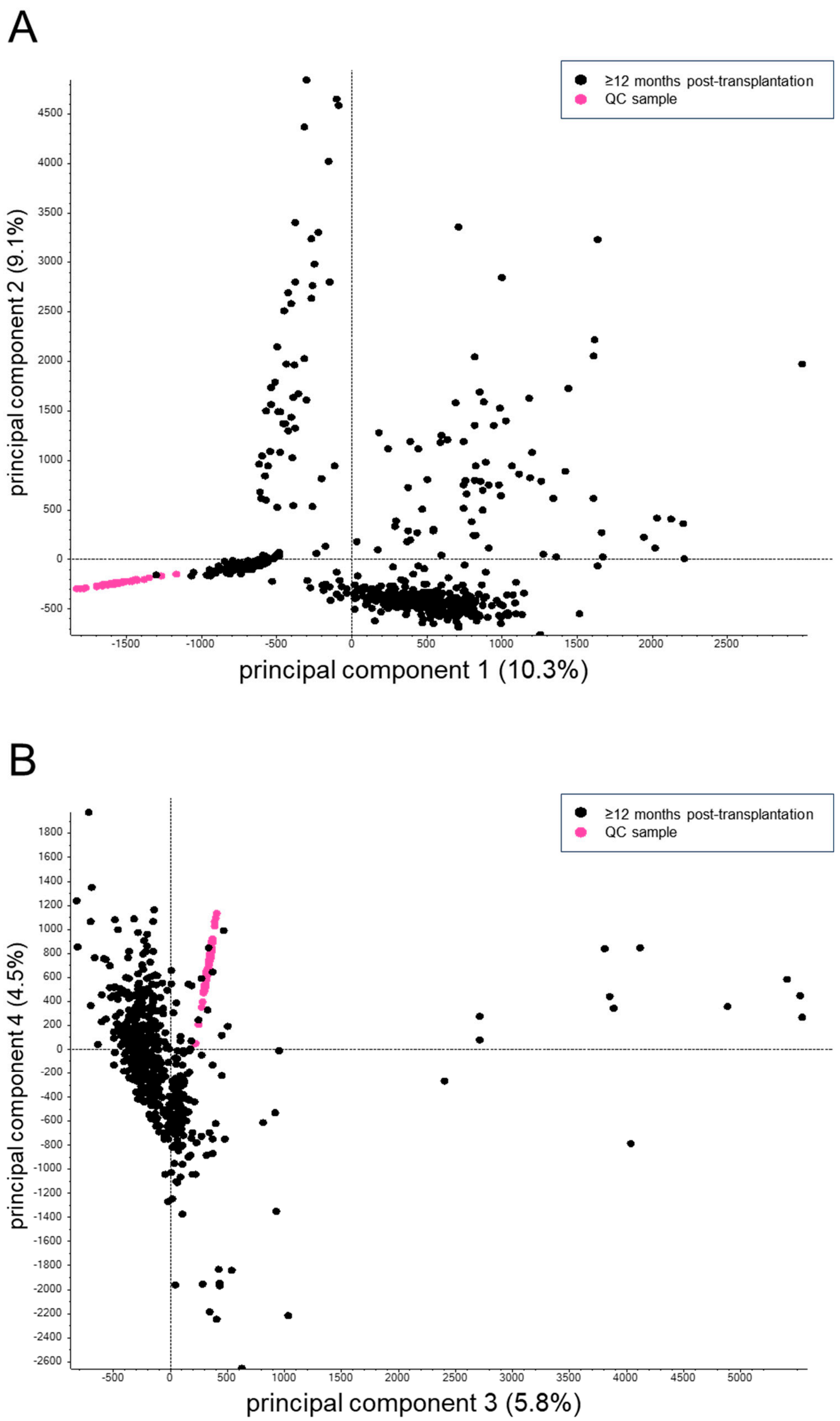

Figure S10. Pareto-scaled scores plots for unsupervised principal component analysis of nonnormalized MS1-level feature data of study A.

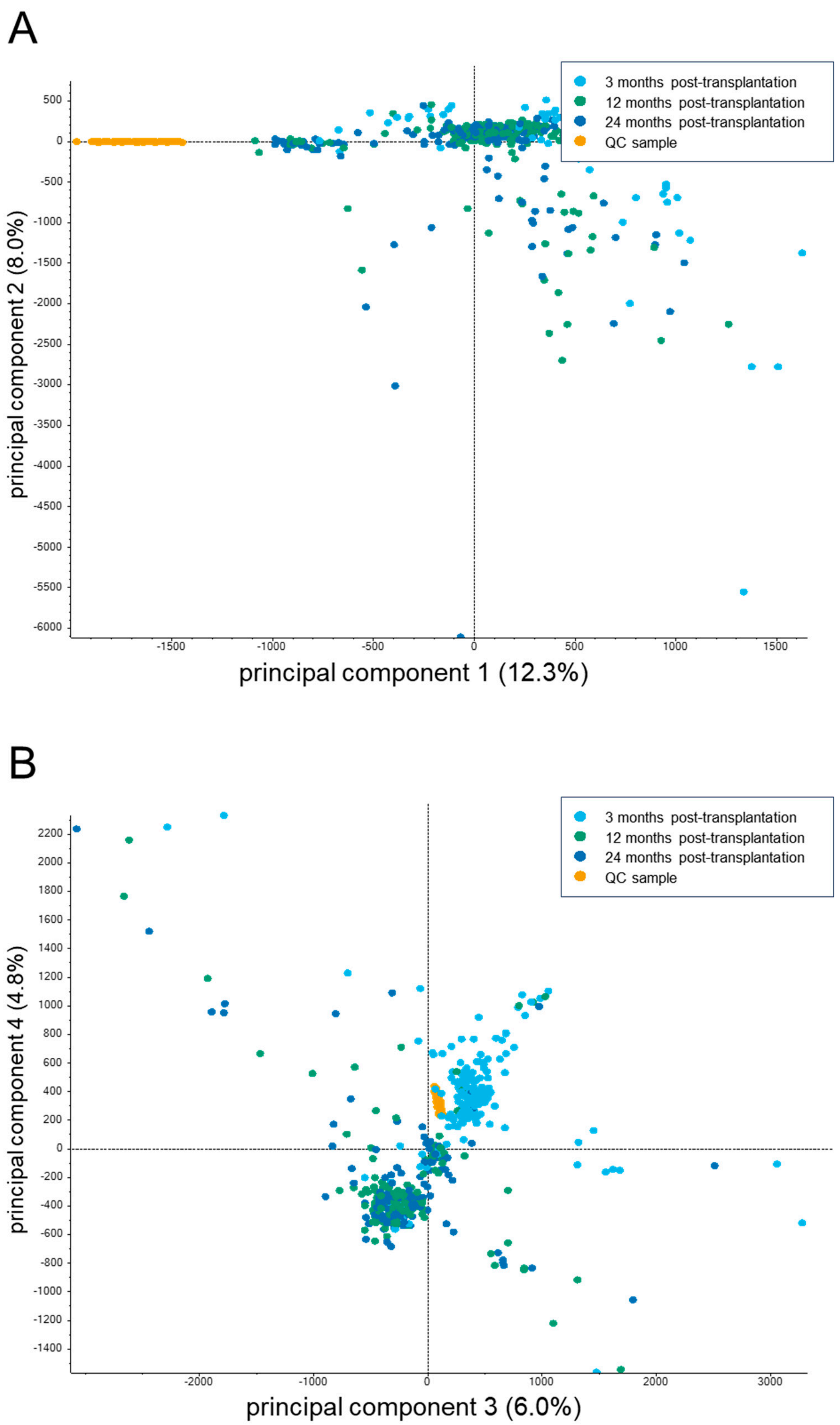

Figure S11. Pareto-scaled scores plots for unsupervised principal component analysis of nonnormalized MS1-level feature data of study B.

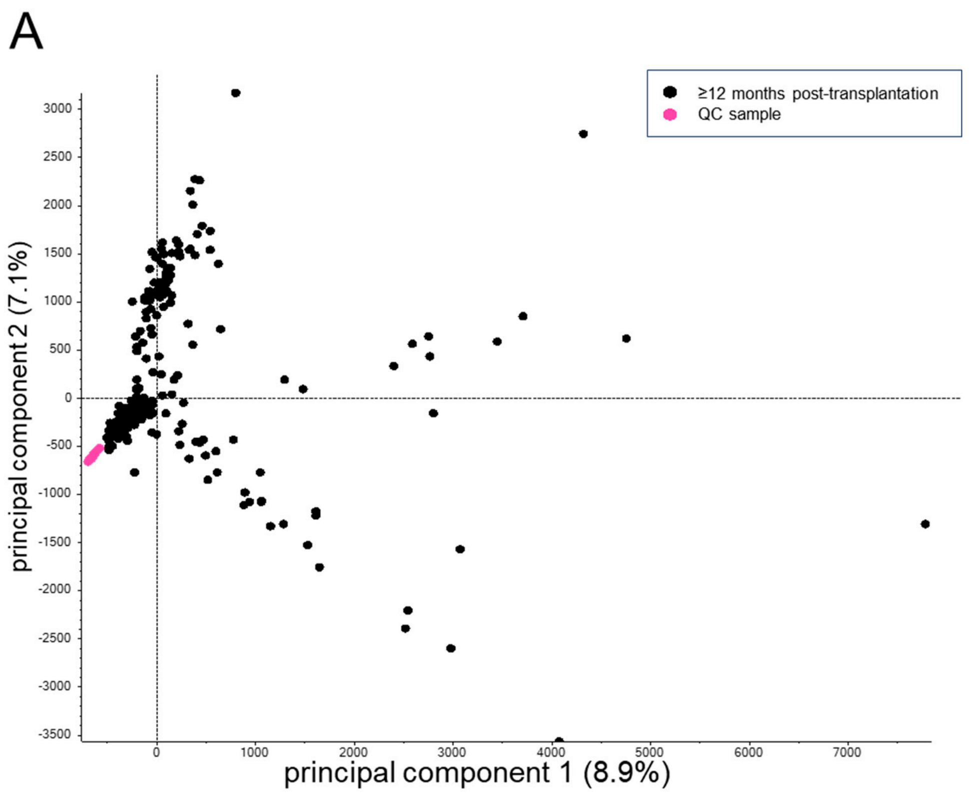

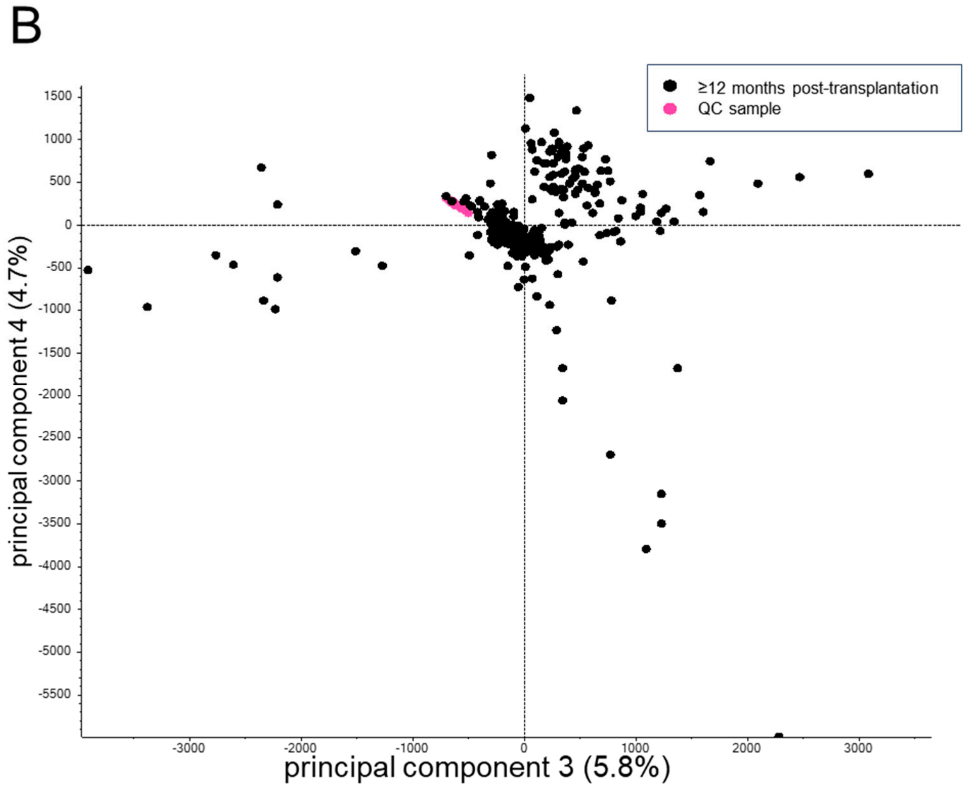

Figure S12. Pareto-scaled scores plots for unsupervised principal component analysis of nonnormalized MS1-level feature data of study C.

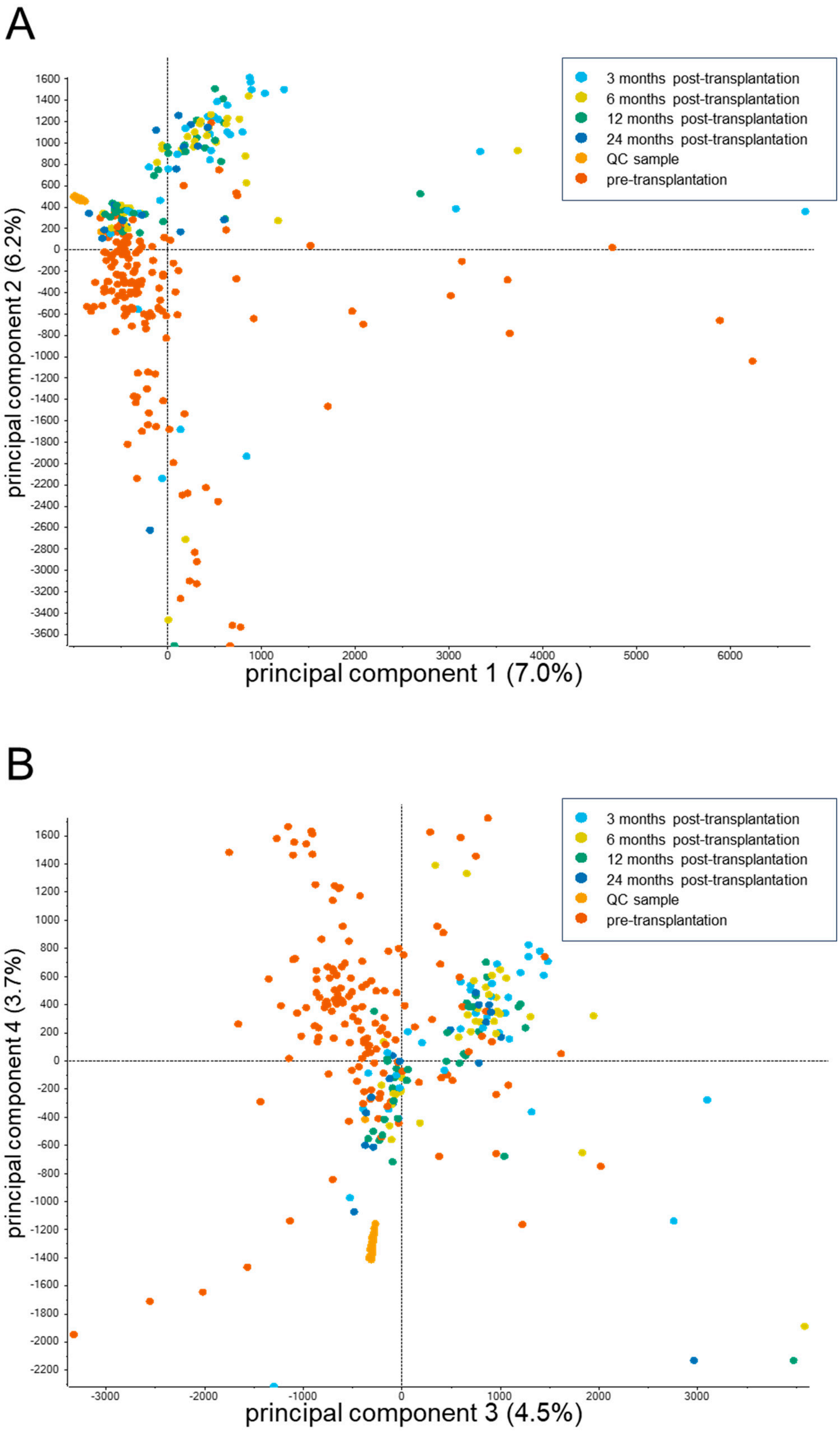

Figure S13. Pareto-scaled scores plots for unsupervised principal component analysis of nonnormalized MS1-level feature data of study D.

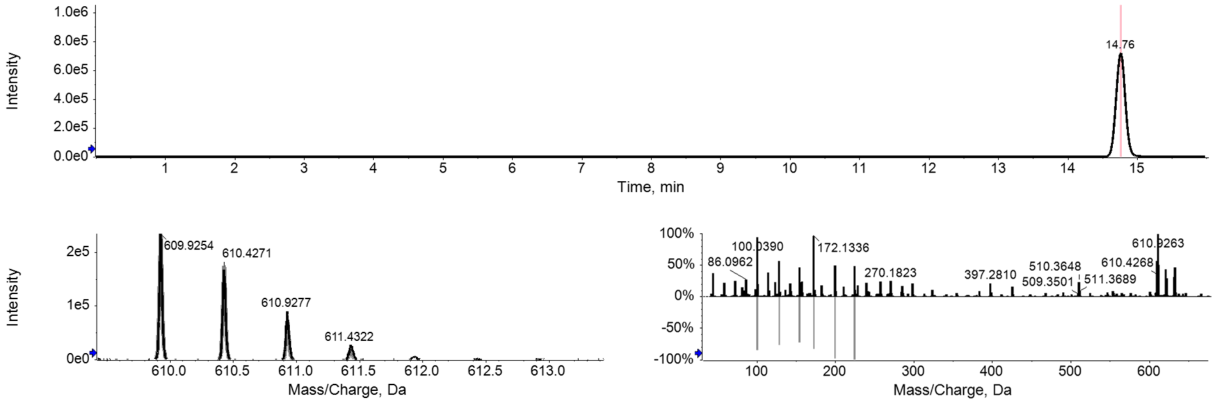

Figure S14. Exemplary spectral library matching-based identification of hydroxylated cyclosporine A (CsA-OH; $[M+2H]^{2+}$; CID 10260788/10653928), as was found in urine of a kidney transplant recipient. All CsA-OH identifications concern 'level 3' metabolite identifications according to the Metabolomics Standards Initiative (Sumner LW, *et al*. Metabolomics 2007; 3: 211–21). Still, we performed spectral library matching as if we were dealing with 'level 1' metabolite identifications by matching our data against a reference spectrum obtained from a commercial cyclosporine A standard (CsA; CID 5284373; Sigma-Aldrich, Cat. No. PHR1092) utilizing the commercial SCIEX PeakView software (version 2.2.0.11391) with the settings presented in supplementary Table S4. However, we modified the extraction mass by changing the molecular formula from $C_{62}H_{111}N_{11}O_{12}$ to $C_{62}H_{111}N_{11}O_{13}$, which gave useful results given that CsA and CsA-OH similar retention and fragmentation behavior in our analytical workflow. Moreover, presumed exposure to CsA could be evaluated more effectively by targeting CsA-OH for which signal intensities levels were typically two to three orders of magnitude greater than those of CsA. The blue and white arrows on the y-axes indicate thresholds for presenting retention time and m/z values.

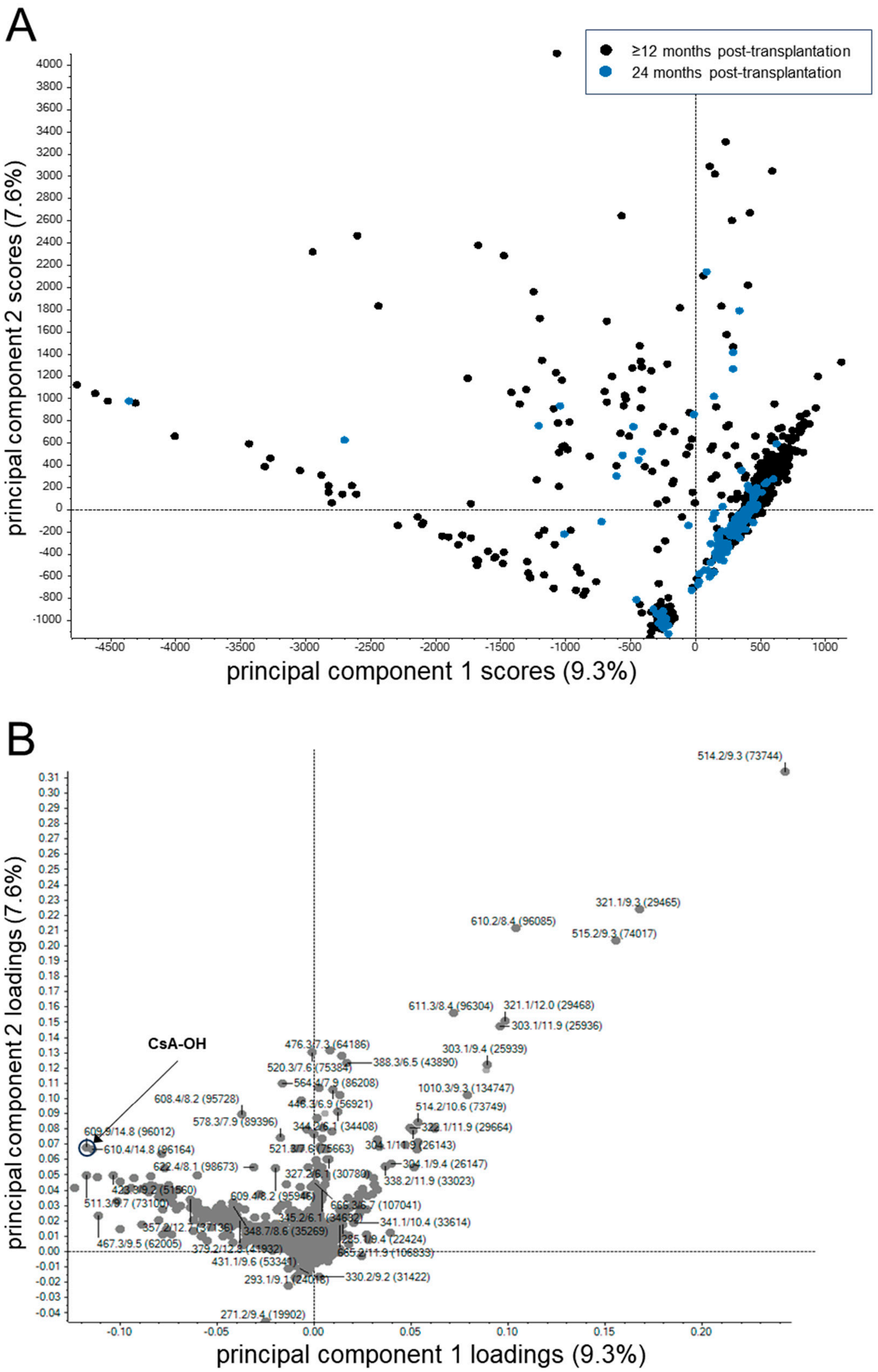

Figure S15. Pareto-scaled (**A**) scores and (**B**) loadings plots for unsupervised principal component analysis of nonnormalized MS1-level feature data of all samples of study A (in black) and the 24 months post-transplantation samples of study B (in blue). In the loadings plot, the feature corresponding to hydroxylated cyclosporine A (CsA-OH; $[M+2H]^{2+}$) is indicated with a blue circle.

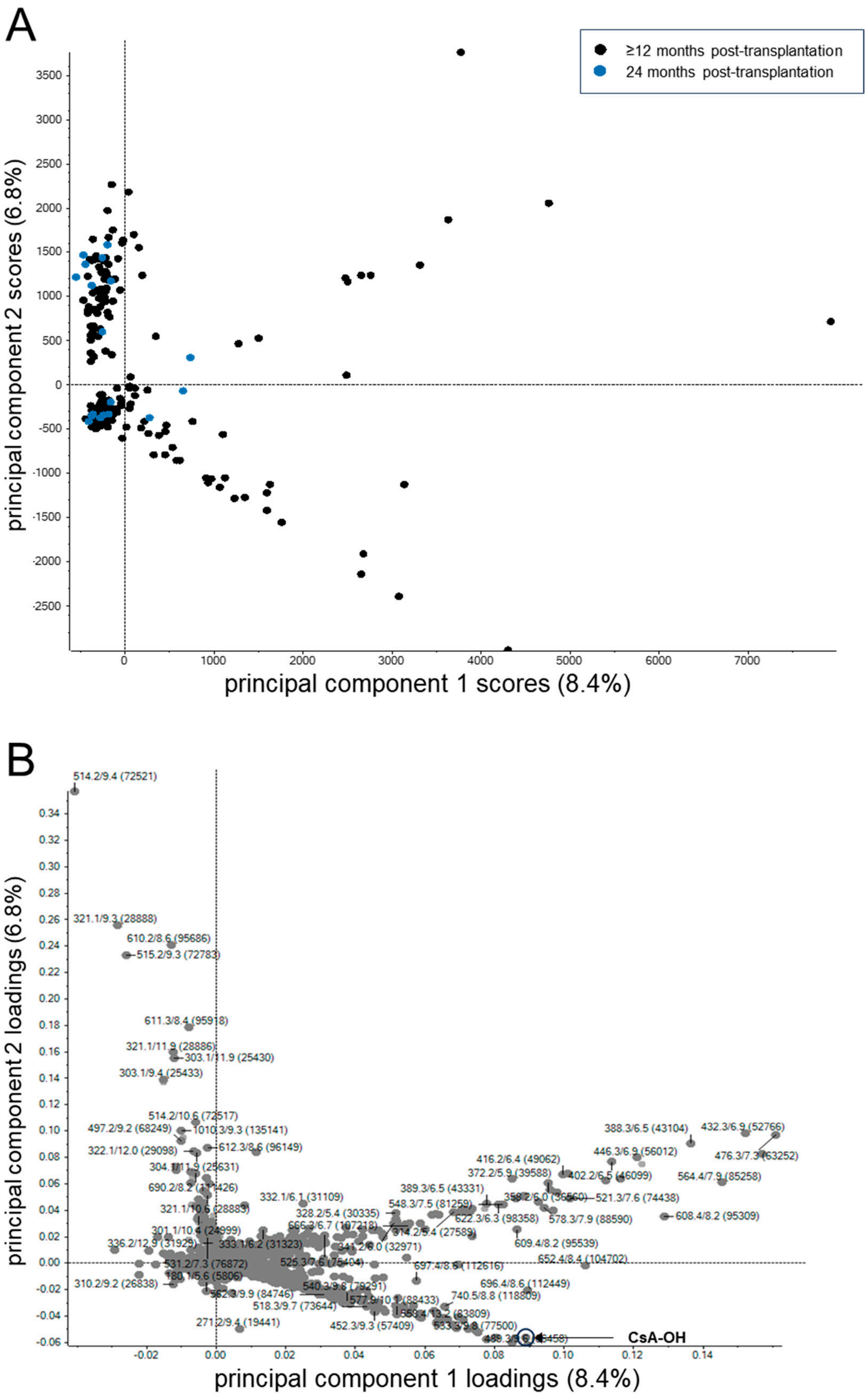

Figure S16. Pareto-scaled (**A**) scores and (**B**) loadings plots for unsupervised principal component analysis of nonnormalized MS1-level feature data of all samples of study C (in black) and the 24 months post-transplantation samples of study D (in blue). In the loadings plot, the feature corresponding to hydroxylated cyclosporine A (CsA-OH; $[M+2H]^{2+}$) is indicated with a blue circle.

A. MS1-level extracted ion chromatogram (m/z 594.9201 ± 0.0025) of an exemplary cyclosporine user

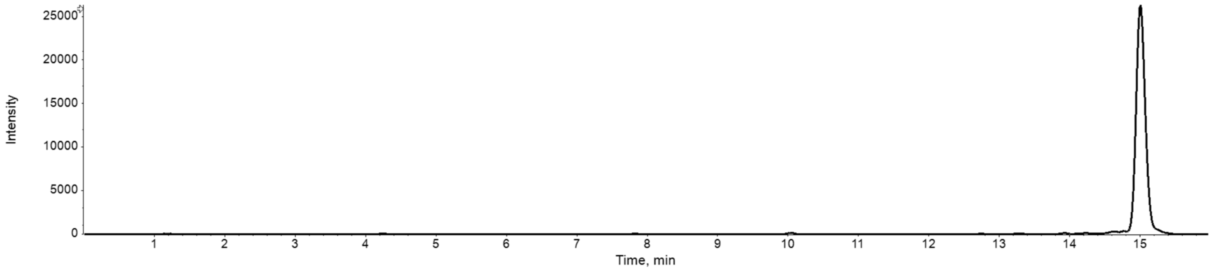

B. SWATH/MS fragment spectrum of the peak at 15.0 min.

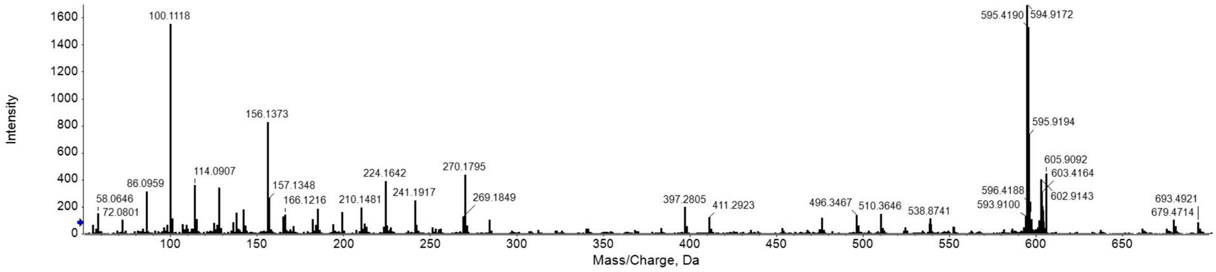

C. Product ion scan fragment spectrum of the peak at 15.0 min.

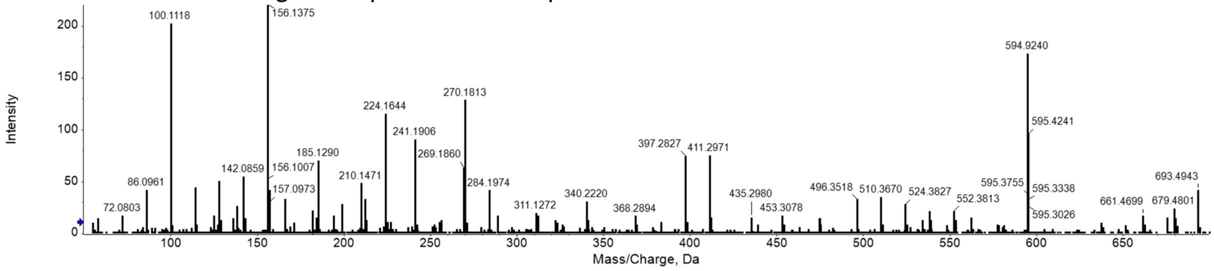

Figure S17. (**A**) MS1-level extracted ion chromatogram, (**B**) SWATH/MS fragment spectrum, and (**C**) product ion scan fragment spectrum of a putatively demethylated version of cyclosporine A (-14 Da) observed in urine of a human cyclosporine A user. The substance featured in this figure (presumably being the metabolite known as 'AM4N' and 'Metabolite 21', CID 11115853) reflects a 'level 3' identification in terms of the classification proposed by the Metabolomics Standards Initiative (Sumner LW, Amberg A, Barrett D, et al. Proposed minimum reporting standards for chemical analysis Chemical Analysis Working Group (CAWG) Metabolomics Standards Initiative (MSI). Metabolomics 2007; 3: 211–221). The blue and white arrows on the y-axes indicate thresholds for presenting retention time and m/z values.

A. MS1-level extracted ion chromatogram (m/z 601.9280 ± 0.0025) of an exemplary cyclosporine user

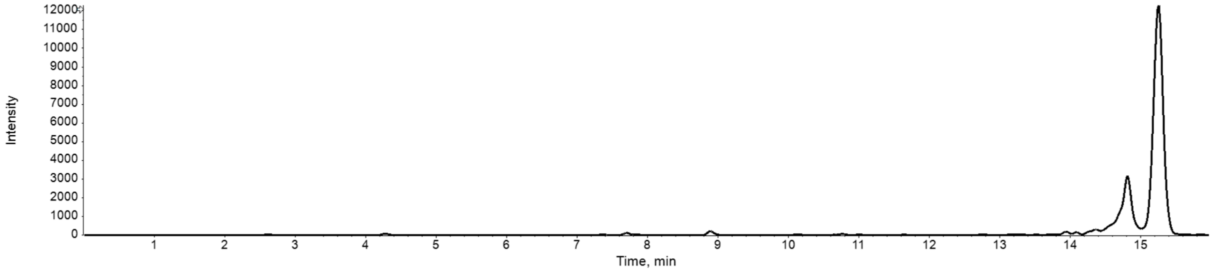

B. SWATH/MS fragment spectrum of the peak at 15.2 min.

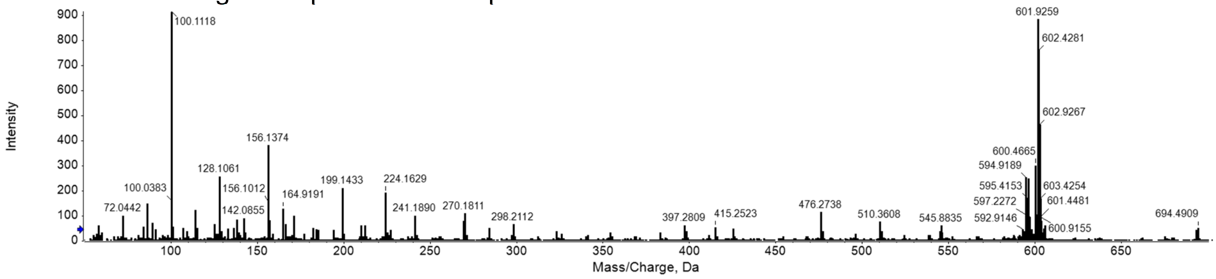

C. Product ion scan fragment spectrum of the peak at 15.2 min.

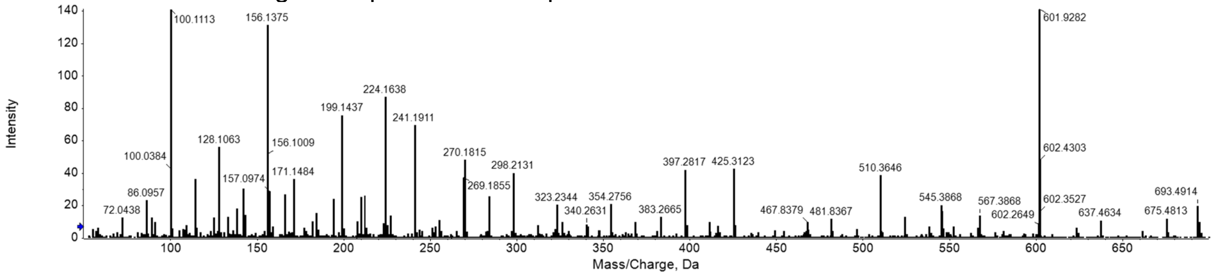

Figure S18. (**A**) MS1-level extracted ion chromatogram, (**B**) SWATH/MS fragment spectrum, and (**C**) product ion scan fragment spectrum of cyclosporine A observed in urine of a human cyclosporine A user. The substance featured in this figure (surely being cyclosporine A based upon confirmation using a commercial chemical reference standard (Sigma-Aldrich, Cat. No. PHR1092), CID 5284373) reflects a 'level 1' identification in terms of the classification proposed by the Metabolomics Standards Initiative (Sumner LW, Amberg A, Barrett D, et al. Proposed minimum reporting standards for chemical analysis Chemical Analysis Working Group (CAWG) Metabolomics Standards Initiative (MSI). Metabolomics 2007; 3: 211–221). The blue and white arrows on the y-axes indicate thresholds for presenting retention time and m/z values.

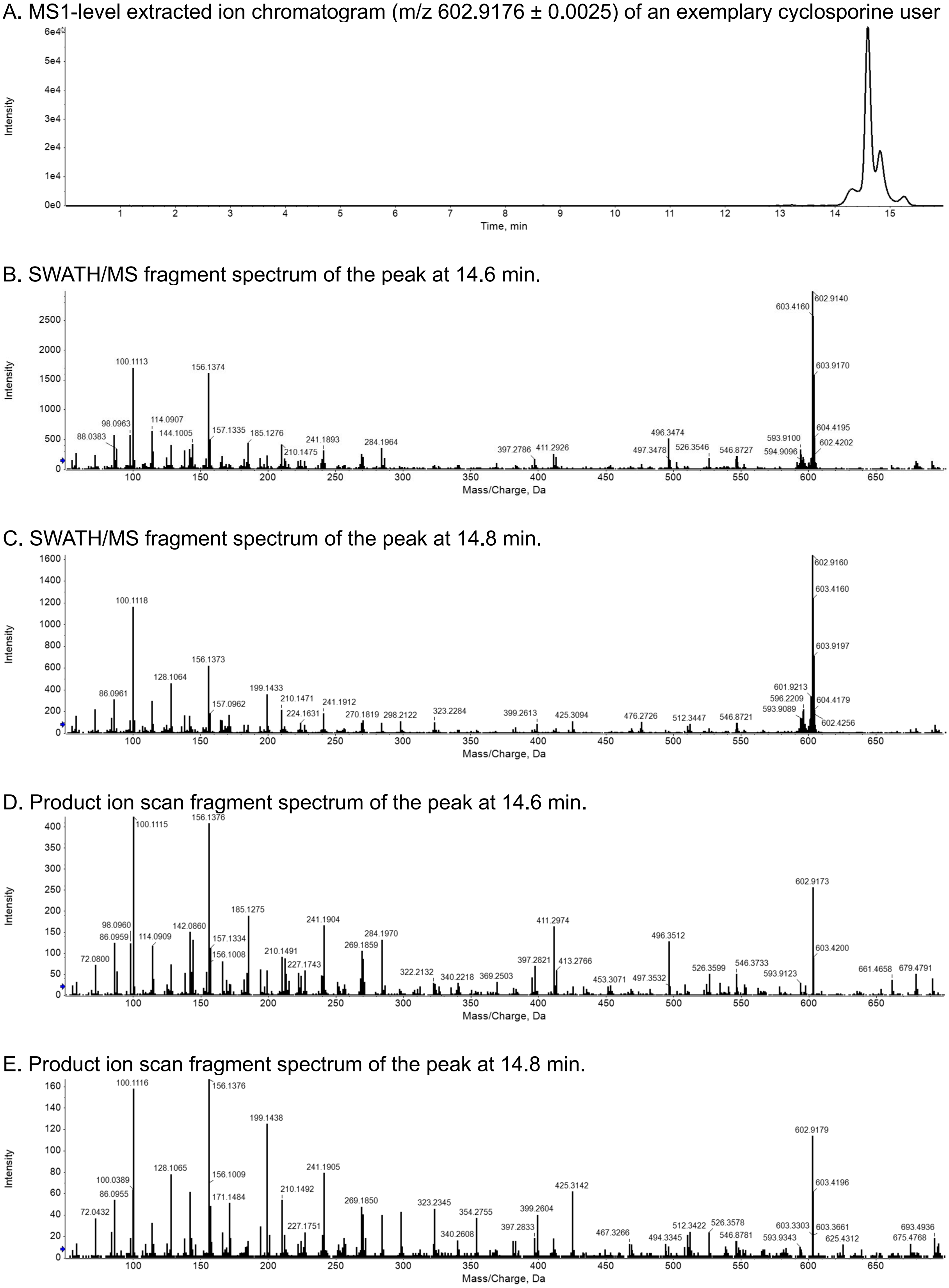

Figure S19. (**A**) MS1-level extracted ion chromatogram, (**B**-**C**) SWATH/MS fragment spectra, and (**D**-**E**) product ion scan fragment spectra of two putatively demethylated and oxygenated versions of cyclosporine A (-14, +16 Da) observed in urine of a human cyclosporine A user. The substances featured in this figure (presumably being the metabolite known as 'AM14N' and 'Metabolite 25', CID 118753624, and the metabolite known as 'AM4N9' and 'Metabolite 13', CID 10887707) reflect 'level 3' identifications in terms of the classification proposed by the Metabolomics Standards Initiative

(Sumner LW, Amberg A, Barrett D, et al. Proposed minimum reporting standards for chemical analysis Chemical Analysis Working Group (CAWG) Metabolomics Standards Initiative (MSI). Metabolomics 2007; 3: 211–221). The blue and white arrows on the y-axes indicate thresholds for presenting retention time and m/z values.

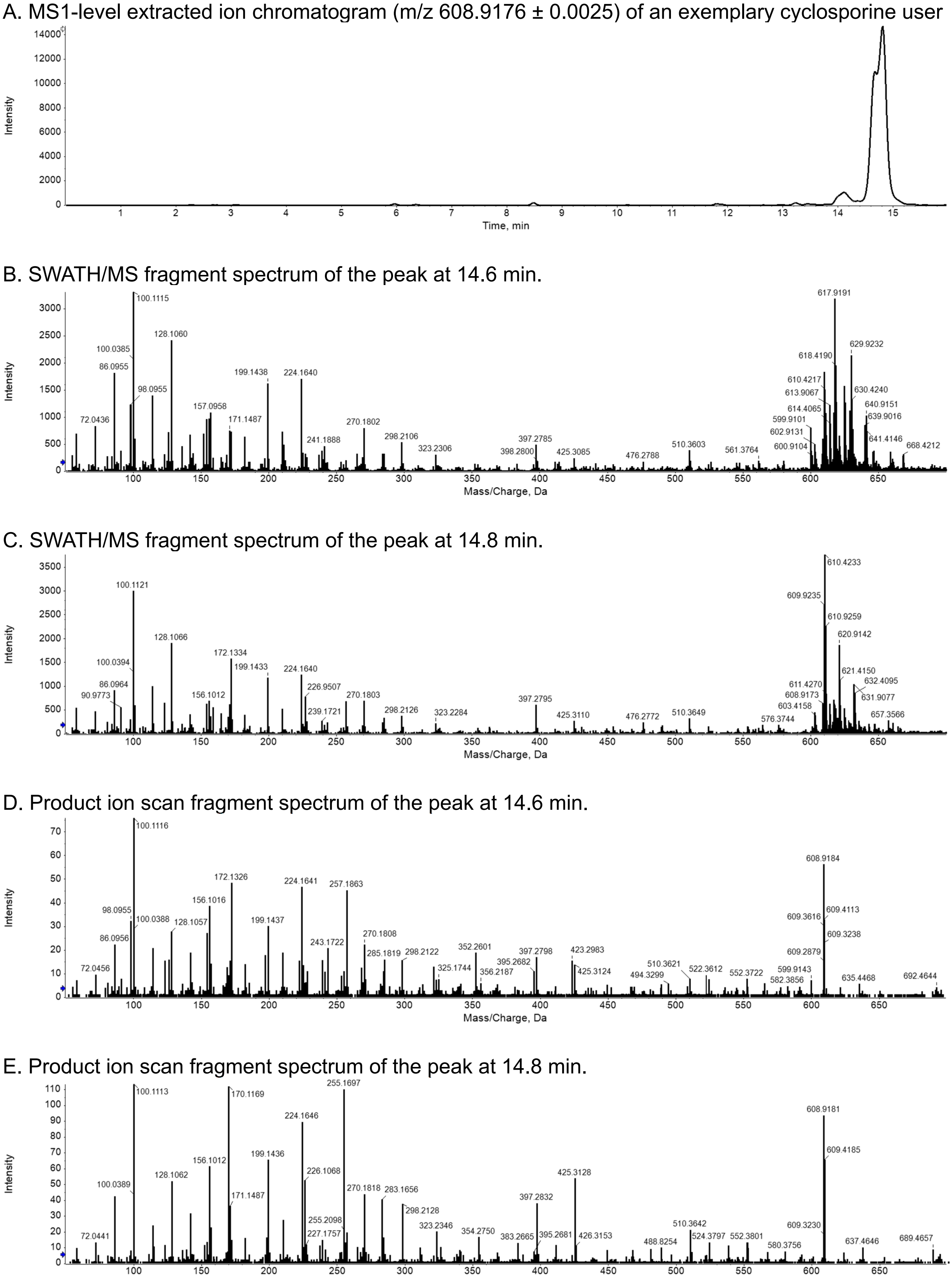

Figure S20. (**A**) MS1-level extracted ion chromatogram, (**B**-**C**) SWATH/MS fragment spectra, and (**D**-**E**) product ion scan fragment spectra of two putatively cyclized, oxygenated, and dehydrogenated versions of cyclosporine A (-2/+2, +16, -2 Da) observed in urine of a human cyclosporine A user. The substances featured in this figure (presumably being the metabolite known as 'AM1cAL' and 'Metabolite 1cAL', CID 132282520, and a differentially oxygenated variant, for which no putative InChI identifier is provided due to the uncertain position of the added functional group) reflect 'level 3'

identifications in terms of the classification proposed by the Metabolomics Standards Initiative (Sumner LW, Amberg A, Barrett D, et al. Proposed minimum reporting standards for chemical analysis Chemical Analysis Working Group (CAWG) Metabolomics Standards Initiative (MSI). Metabolomics 2007; 3: 211–221). The blue and white arrows on the y-axes indicate thresholds for presenting retention time and m/z values.

A. MS1-level extracted ion chromatogram (m/z 609.9254 ± 0.0025) of an exemplary cyclosporine user

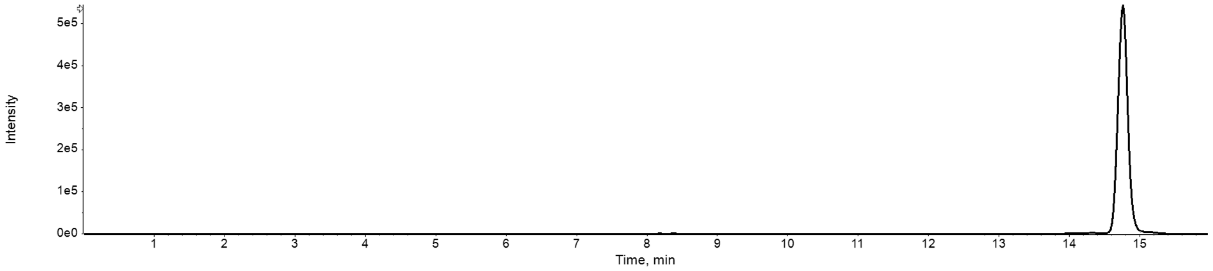

B. SWATH/MS fragment spectrum of the peak at 14.8 min.

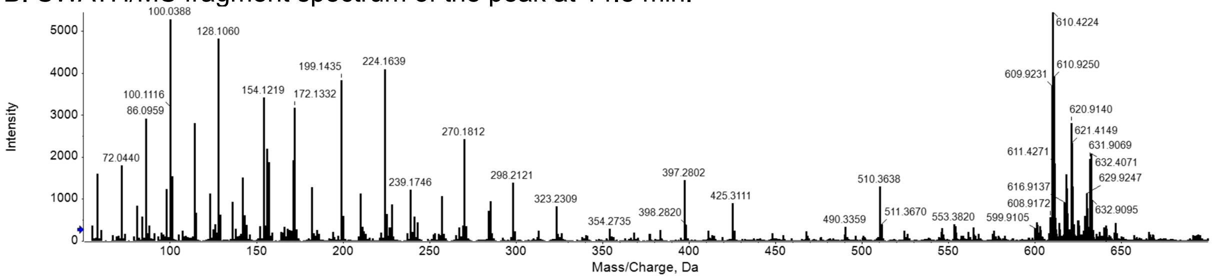

C. Product ion scan fragment spectrum of the peak at 14.8 min.

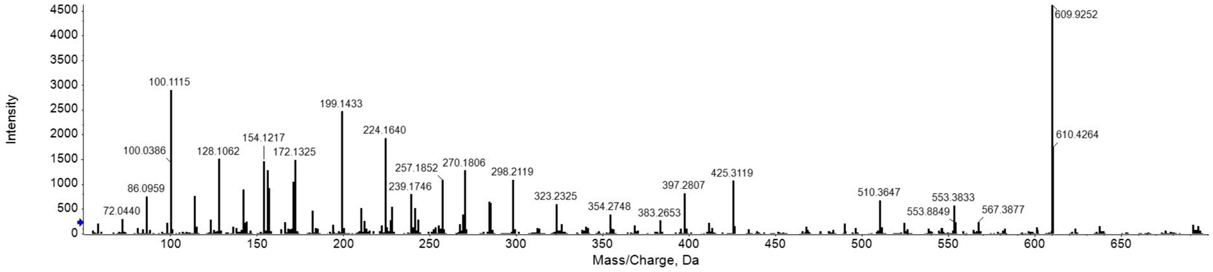

Figure S21. (**A**) MS1-level extracted ion chromatogram, (**B**) SWATH/MS fragment spectrum, and (**C**) product ion scan fragment spectrum of a putatively oxygenated version of cyclosporine A (+16 Da) observed in urine of a human cyclosporine A user. The substance featured in this figure (presumably being the metabolite known as 'AM1' and 'Metabolite 17', CID 10653928, or the metabolite known as 'AM9' and 'Metabolite 1', CID 10260788) reflects a 'level 3' identification in terms of the classification proposed by the Metabolomics Standards Initiative (Sumner LW, Amberg A, Barrett D, et al. Proposed minimum reporting standards for chemical analysis Chemical Analysis Working Group (CAWG) Metabolomics Standards Initiative (MSI). Metabolomics 2007; 3: 211–221). The blue and white arrows on the y-axes indicate thresholds for presenting retention time and m/z values.

A. MS1-level extracted ion chromatogram (m/z 610.9151 ± 0.0025) of an exemplary cyclosporine user

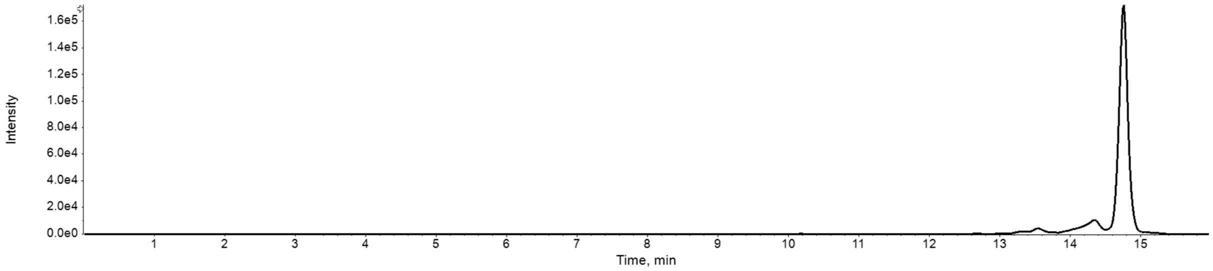

B. SWATH/MS fragment spectrum of the peak at 14.3 min.

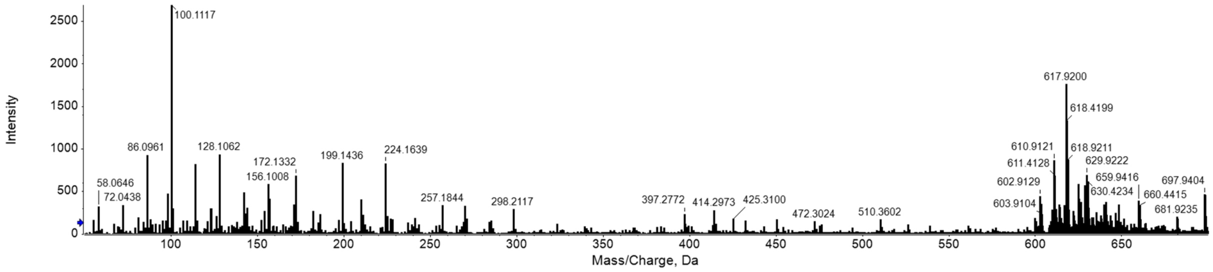

C. Product ion scan fragment spectrum of the peak at 14.3 min.

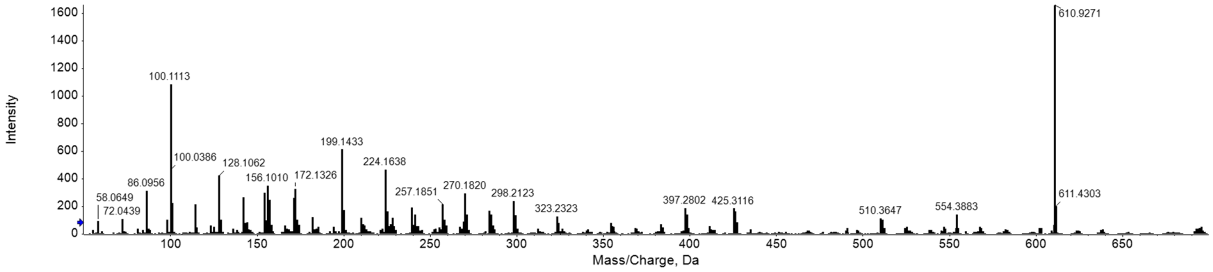

Figure S22. (**A**) MS1-level extracted ion chromatogram, (**B**) SWATH/MS fragment spectrum, and (**C**) product ion scan fragment spectrum of a putatively demethylated and doubly oxygenated version of cyclosporine A (-14, +32 Da) observed in urine of a human cyclosporine A user. The substance featured in this figure (presumably being the metabolite known as 'AM4N69' and 'Metabolite 9', CID 118753622) reflects a 'level 3' identification in terms of the classification proposed by the Metabolomics Standards Initiative (Sumner LW, Amberg A, Barrett D, et al. Proposed minimum reporting standards for chemical analysis Chemical Analysis Working Group (CAWG) Metabolomics Standards Initiative (MSI). Metabolomics 2007; 3: 211–221). The blue and white arrows on the y-axes indicate thresholds for presenting retention time and m/z values.

A. MS1-level extracted ion chromatogram (m/z 616.9151 ± 0.0025) of an exemplary cyclosporine user

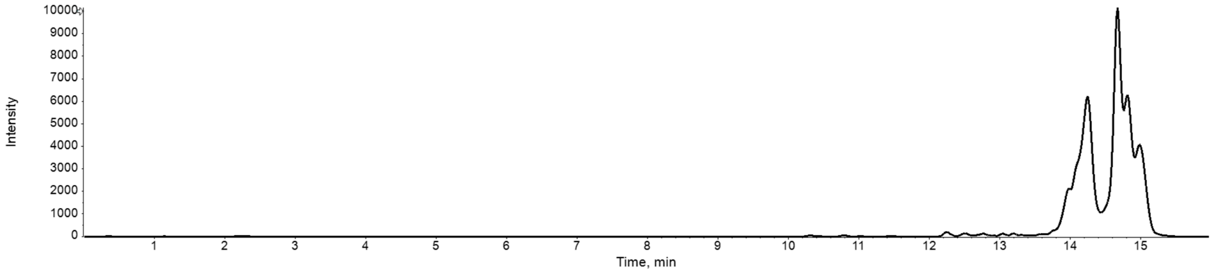

B. SWATH/MS fragment spectrum of the peak at 14.6 min.

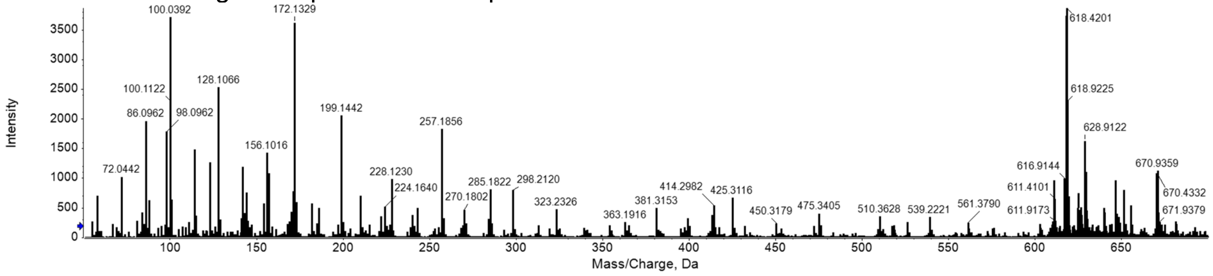

C. Product ion scan fragment spectrum of the peak at 14.6 min.

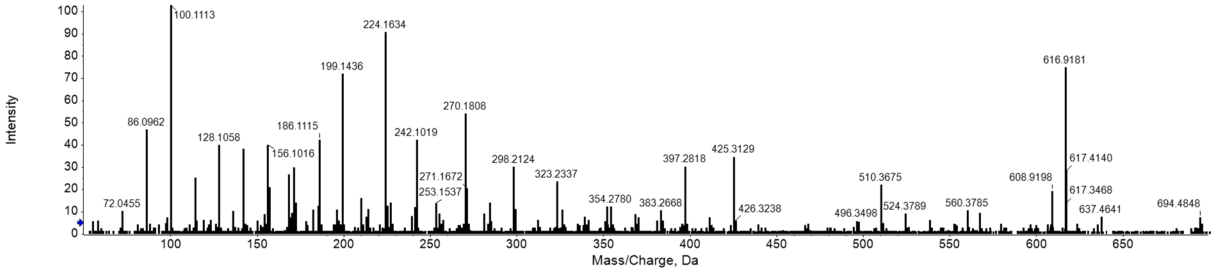

Figure S23. (**A**) MS1-level extracted ion chromatogram, (**B**) SWATH/MS fragment spectrum, and (**C**) product ion scan fragment spectrum of a putatively carboxylated version of cyclosporine A (+32/-2 Da) observed in urine of a human cyclosporine A user. The substance featured in this figure (presumably being the metabolite known as 'AM1A' and 'Metabolite 203-218', CID 86666680) reflects a 'level 3' identification in terms of the classification proposed by the Metabolomics Standards Initiative (Sumner LW, Amberg A, Barrett D, et al. Proposed minimum reporting standards for chemical analysis Chemical Analysis Working Group (CAWG) Metabolomics Standards Initiative (MSI). Metabolomics 2007; 3: 211–221). The blue and white arrows on the y-axes indicate thresholds for presenting retention time and m/z values.

A. MS1-level extracted ion chromatogram (m/z 617.9229 ± 0.0025) of an exemplary cyclosporine user

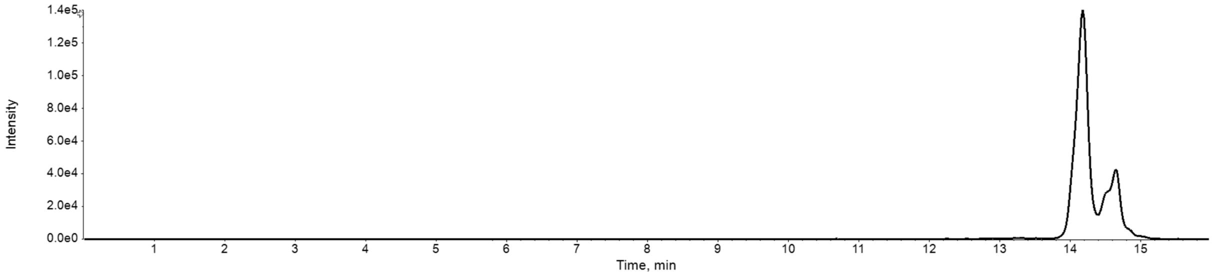

B. SWATH/MS fragment spectrum of the peak at 14.2 min.

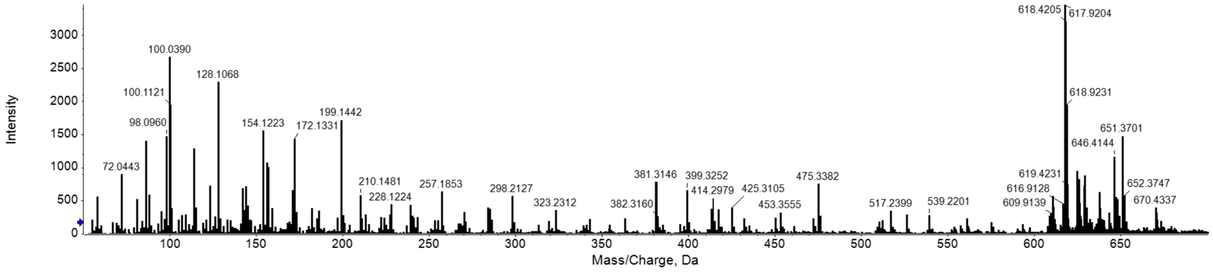

C. SWATH/MS fragment spectrum of the peak at 14.7 min.

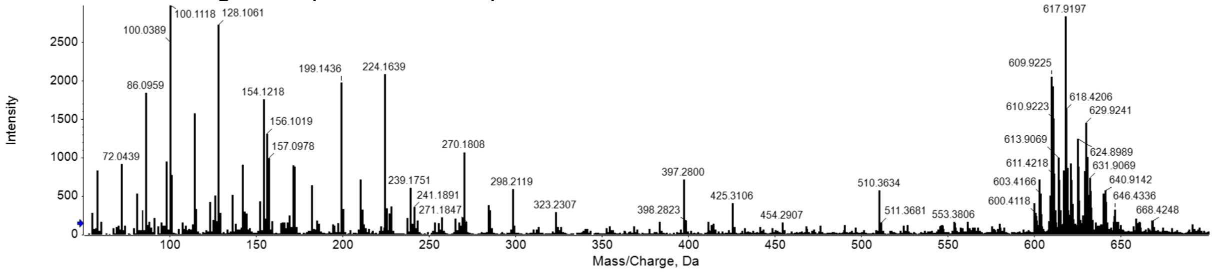

D. Product ion scan fragment spectrum of the peak at 14.2 min.

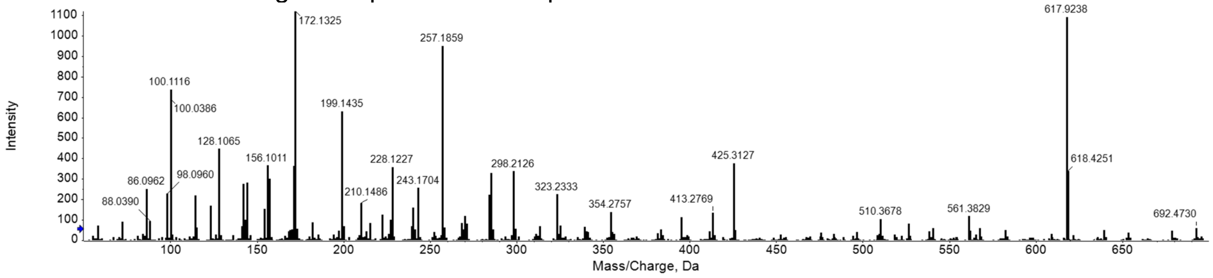

E. Product ion scan fragment spectrum of the peak at 14.7 min.

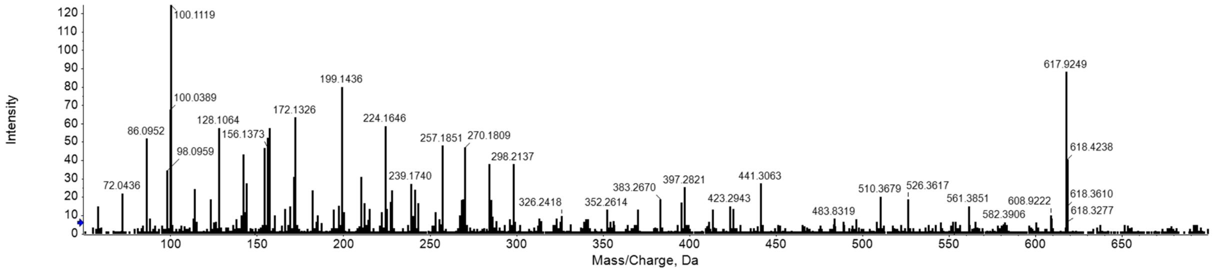

Figure S24. (**A**) MS1-level extracted ion chromatogram, (**B**-**C**) SWATH/MS fragment spectra, and (**D**-**E**) product ion scan fragment spectra of two putatively doubly oxygenated versions of cyclosporine A (+32 Da) observed in urine of a human cyclosporine A user. The substances featured in this figure (presumably being the metabolite known as 'AM19' and 'Metabolite 8', CID 102089122, and/or the metabolite known as 'AM49' and 'Metabolite 10', CID 10192176, and/or the metabolite known as

‘AM69’ and ‘Metabolite 16’, CID 118753621, and/or the metabolite known as ‘AM1c9’ and ‘Metabolite 26’, CID 118753574) reflect ‘level 3’ identifications in terms of the classification proposed by the Metabolomics Standards Initiative (Sumner LW, Amberg A, Barrett D, et al. Proposed minimum reporting standards for chemical analysis Chemical Analysis Working Group (CAWG) Metabolomics Standards Initiative (MSI). Metabolomics 2007; 3: 211–221). The blue and white arrows on the y-axes indicate thresholds for presenting retention time and m/z values.

A. MS1-level extracted ion chromatogram (m/z 624.9125 ± 0.0025) of an exemplary cyclosporine user

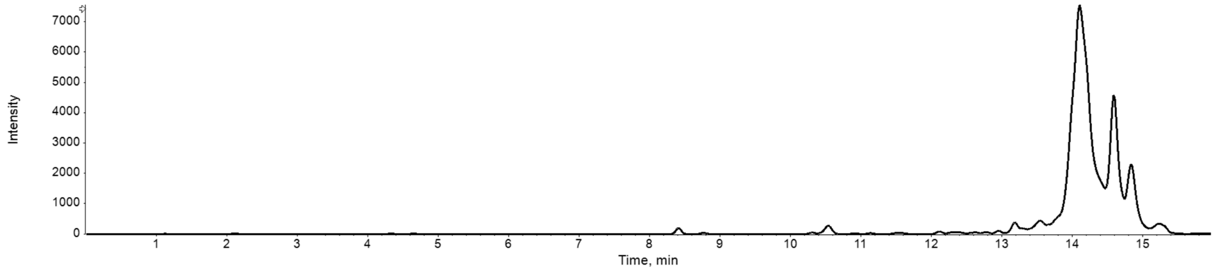

B. SWATH/MS fragment spectrum of the peak at 14.2 min.

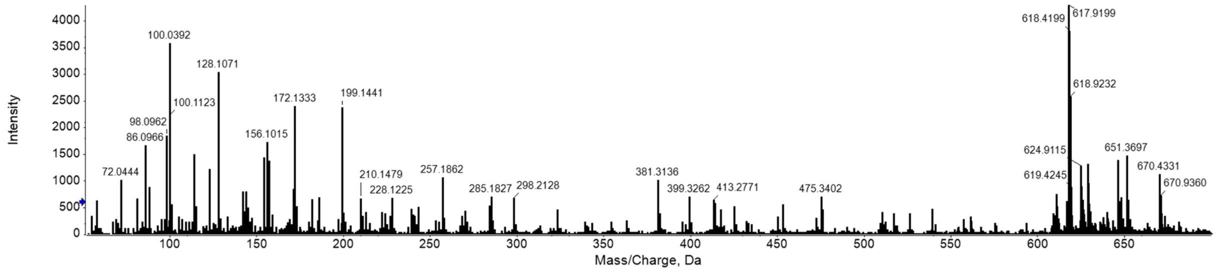

C. Product ion scan fragment spectrum of the peak at 14.2 min.

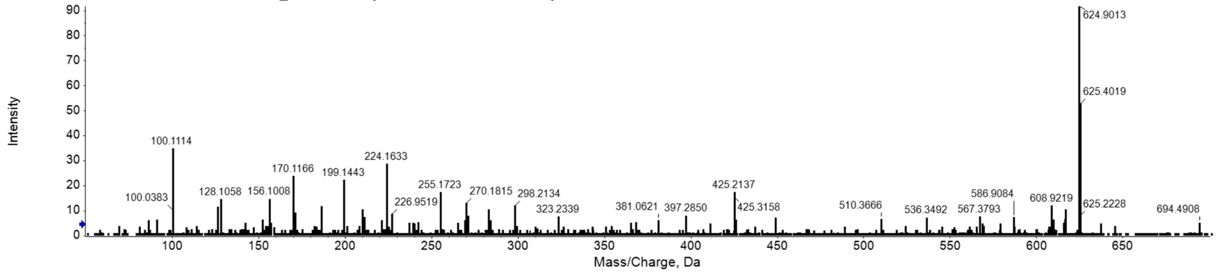

Figure S25. (**A**) MS1-level extracted ion chromatogram, (**B**) SWATH/MS fragment spectrum, and (**C**) product ion scan fragment spectrum of a putatively oxygenated and carboxylated version of cyclosporine A (+16, +32/-2 Da) observed in urine of a human cyclosporine A user. The substance featured in this figure (presumably being a further oxygenated version of the metabolite known as 'AM1A' and 'Metabolite 203-218', for which no putative InChI identifier is provided due to the uncertain position of the added functional group) reflects a 'level 3' identification in terms of the classification proposed by the Metabolomics Standards Initiative (Sumner LW, Amberg A, Barrett D, et al. Proposed minimum reporting standards for chemical analysis Chemical Analysis Working Group (CAWG) Metabolomics Standards Initiative (MSI). Metabolomics 2007; 3: 211–221). The blue and white arrows on the y-axes indicate thresholds for presenting retention time and m/z values.

A. MS1-level extracted ion chromatogram (m/z 670.4336 ± 0.0025) of an exemplary cyclosporine user

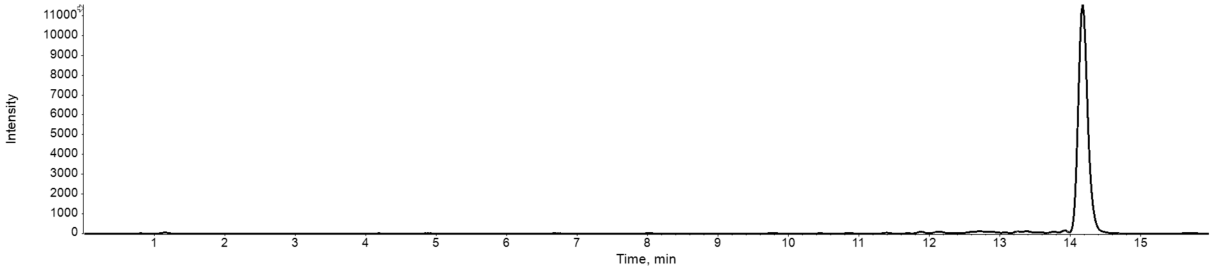

B. SWATH/MS fragment spectrum of the peak at 14.1 min.

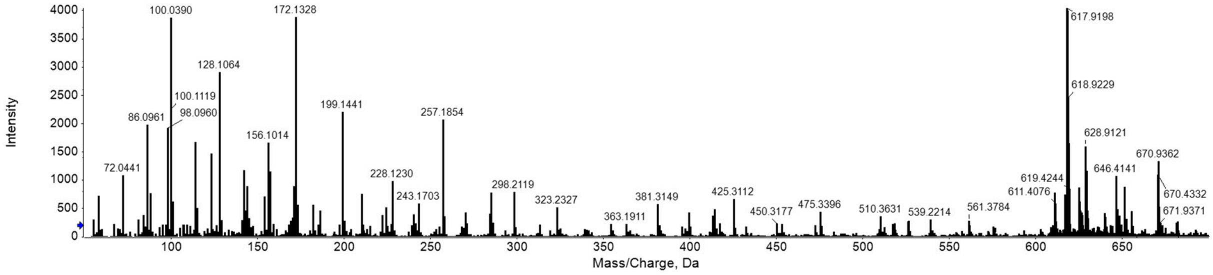

C. Product ion scan fragment spectrum of the peak at 14.1 min.

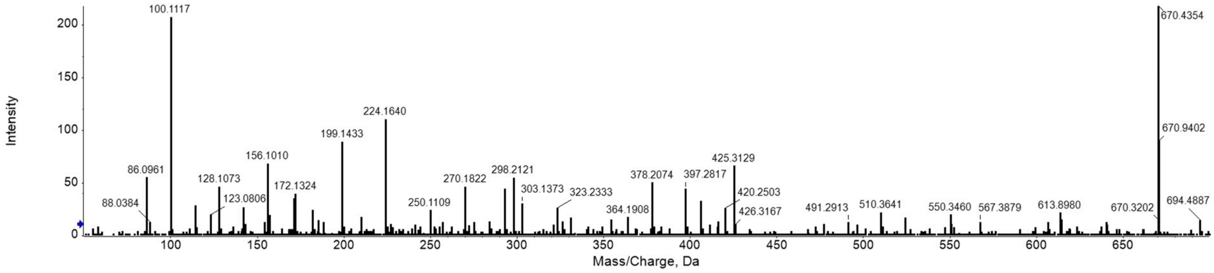

Figure S26. (**A**) MS1-level extracted ion chromatogram, (**B**) SWATH/MS fragment spectrum, and (**C**) product ion scan fragment spectrum of a putative product of oxygenation and glutathione conjugation of cyclosporine A observed in urine of a human cyclosporine A user. The substance featured in this figure (presumably being a product of oxygenation and glutathione conjugation, for which no putative InChI identifier is provided due to the uncertain identity of the added functional group) reflects a 'level 3' identification in terms of the classification proposed by the Metabolomics Standards Initiative (Sumner LW, Amberg A, Barrett D, et al. Proposed minimum reporting standards for chemical analysis Chemical Analysis Working Group (CAWG) Metabolomics Standards Initiative (MSI). Metabolomics 2007; 3: 211–221). The blue and white arrows on the y-axes indicate thresholds for presenting retention time and m/z values.
